# Increased response of postmenopausal bone to interval walking training depends on baseline bone mineral density

**DOI:** 10.1371/journal.pone.0309936

**Published:** 2024-09-05

**Authors:** Rizka Nugraheni Martyanti, Mayuko Morikawa, Masaaki Hanaoka, Satoshi Tanaka, Yukio Nakamura, Hiroshi Nose, Shizue Masuki

**Affiliations:** 1 Departments of Sports Medical Sciences, Shinshu University Graduate School of Medicine, Nagano, Japan; 2 Department of Anesthesiology and Resuscitology, Shinshu University Graduate School of Medicine, Nagano, Japan; 3 Institute for Biomedical Sciences, Shinshu University, Nagano, Japan; 4 Jukunen Taiikudaigaku Research Center, Nagano, Japan; 5 Department of e-Health Sciences, Shinshu University Graduate School of Medicine, Nagano, Japan; 6 Department of Orthopaedic Surgery, Shinshu University Graduate School of Medicine, Nagano, Japan; Ehime University Graduate School of Medicine, JAPAN

## Abstract

**Purpose:**

To examine the hypothesis that an increase in response of postmenopausal bone to interval walking training (IWT) depends on baseline bone mineral densities (BMDs).

**Methods:**

Two hundred and thirty-four postmenopausal women (64±5 (SD) yr) with no medication for osteoporosis performed 5-month IWT, repeating fast and slow walking at ≥70% and ~40% peak aerobic capacity, respectively, for 3 minutes each per set, ≥5 sets/day, ≥4 days/week. They were recruited from those who had performed IWT ≥6 months before participating in the study so that their physical fitness and lifestyle-related disease symptoms had almost reached a steady state at the time of their participation. We measured BMDs for the lumbar spine (LS), bilateral femoral neck (FN), and bilateral total hip (TH) by dual-energy X-ray absorptiometry (DXA) before and after the intervention. We used a multiple regression analysis to identify significant independent factors for increasing BMDs after the intervention as baseline physical characteristics, exercise intensity, and exercise time during IWT were the candidates. For any bone site where the independent factor identified was singular, we analyzed the relationship between the identified factor vs increases in BMD after the intervention (ΔBMD) by the locally weighted scatterplot smoothing (LOWESS) method.

**Results:**

Almost all subjects completed the designated protocol with minimal adverse events. We found that significant determinants for increasing BMDs were the baseline BMDs for all bone sites, as well as age and body mass index for TH (all, P<0.02). Furthermore, the LOWESS trendline between ΔBMDs vs the baseline BMDs, divided equally into 10 bins for LS and FN, respectively, showed that ΔBMD responses (*Y*) were attenuated as the baseline BMDs (*X*) increased in the lower 4 bins and then showed a flat line (*Y* = ~0) in the remaining higher 6 bins for LS and FN. When the lower 4 bins and the higher 6 bins of the baseline BMD were pooled, respectively, BMDs significantly increased by 1.8% and 1.0% in the lower groups for LS and FN, respectively (both, P<0.001) while not in the higher groups after the intervention (both, P>0.3).

**Conclusions:**

IWT may be of benefit with minimal adverse events to postmenopausal women, although the effects were greater in those with lower baseline BMDs.

**Trial registration:**

UMIN000047428. https://rctportal.niph.go.jp/s/detail/um?trial_id=UMIN000047428#.

## Introduction

Continuous growth in the elderly population increases the prevalence of osteoporosis, especially in postmenopausal women, as aging accelerates bone loss progressively by 0.6%, 1.1%, and 2.1% per year for the 60–69, 70–79, and >80 age groups, respectively [1, 2]. Furthermore, the loss in bone mineral density (BMD) increases the risk of fractures leading to impaired quality of life, disability, and mortality [3]. To prevent these issues, a training regimen composed of aerobic, resistance, and flexibility exercise has been widely recommended as a non-pharmacological method by the American College of Sports Medicine (ACSM) [4], and walking is one of the most popular aerobic training regimens for older people because it is relatively safe and easy to perform [5]. However, due to methodological limitations [6], there have been a limited number of studies to examine the effects of aerobic training while considering baseline physical characteristics [7], intensity [8], and volume (time) [9] of the training in a large population of subjects.

In the present study, we used the “e-Health Promotion System”, which we have developed [10] for a large population of middle-aged and older people to perform walking training. The system consists of interval walking training (IWT) and an Internet of Things (IoT) platform for monitoring exercise intensity and volume (time) during IWT. The IWT is a training regimen repeating ≥5 sets of fast walking at ≥70% individual peak aerobic capacity (V̇O_2peak_) and slow walking at ~40% V̇O_2peak_ for 3 minutes each and ≥4 days/week, during which exercise intensity (energy expenditure per min) is measured with a portable calorimeter equipped with a tri-axial accelerometer and barometer. We used the aerobic exercise intensity to estimate the strain magnitude on the bones, assuming that the strain would increase as the exercise intensity relative to individual V̇O_2peak_ increased.

The reason for adopting the relative exercise intensity for walking training was based on the assumption that baseline BMD would increase in subjects with higher V̇O_2peak_ so that greater excise intensity would be needed to increase BMD from the “mechanostat” theory [11]. Although these assumptions have been generally accepted and have led to the broad recommendation of moderate- to high-intensity aerobic exercise for individuals to prevent osteoporosis by ACSM [4], they might not always be correct because aerobic stress to cardio-respiratory function and mechanical strain to bones during exercise are essentially different, and therefore, we postulated that the strain on the bones during IWT would not meet the strain required to increase BMD as individual V̇O_2peak_ increased.

Based on these assumptions, the main aim of the present study was to examine the hypothesis that an increase in response of the lumbar spine (LS), bilateral femoral neck (FN), and bilateral total hip (TH) bones to IWT in menopausal women depends on the baseline BMDs but with upper limits for the responses. If the hypothesis proved correct in any bone site without other confounding effects, a second aim of the present study was then to precisely analyze the relationships between the baseline BMDs vs the increase in BMDs and to consider any underlying mechanisms for this response.

To minimize any influence of baseline physical characteristics other than BMD, such as physical fitness, physical activity, and lifestyle-related disease symptoms before the intervention and their changes on bone formation during the intervention [12–17], we recruited subjects from those who had performed IWT ≥6 months before participating in the study where other baseline variables had reportedly reached a steady state [18]. By using these subjects and considering these potential influences, we thought that we could examine any specific effects of mechanical strain on bone formation induced by IWT.

## Materials and methods

### Trial design

**Fig 1:**
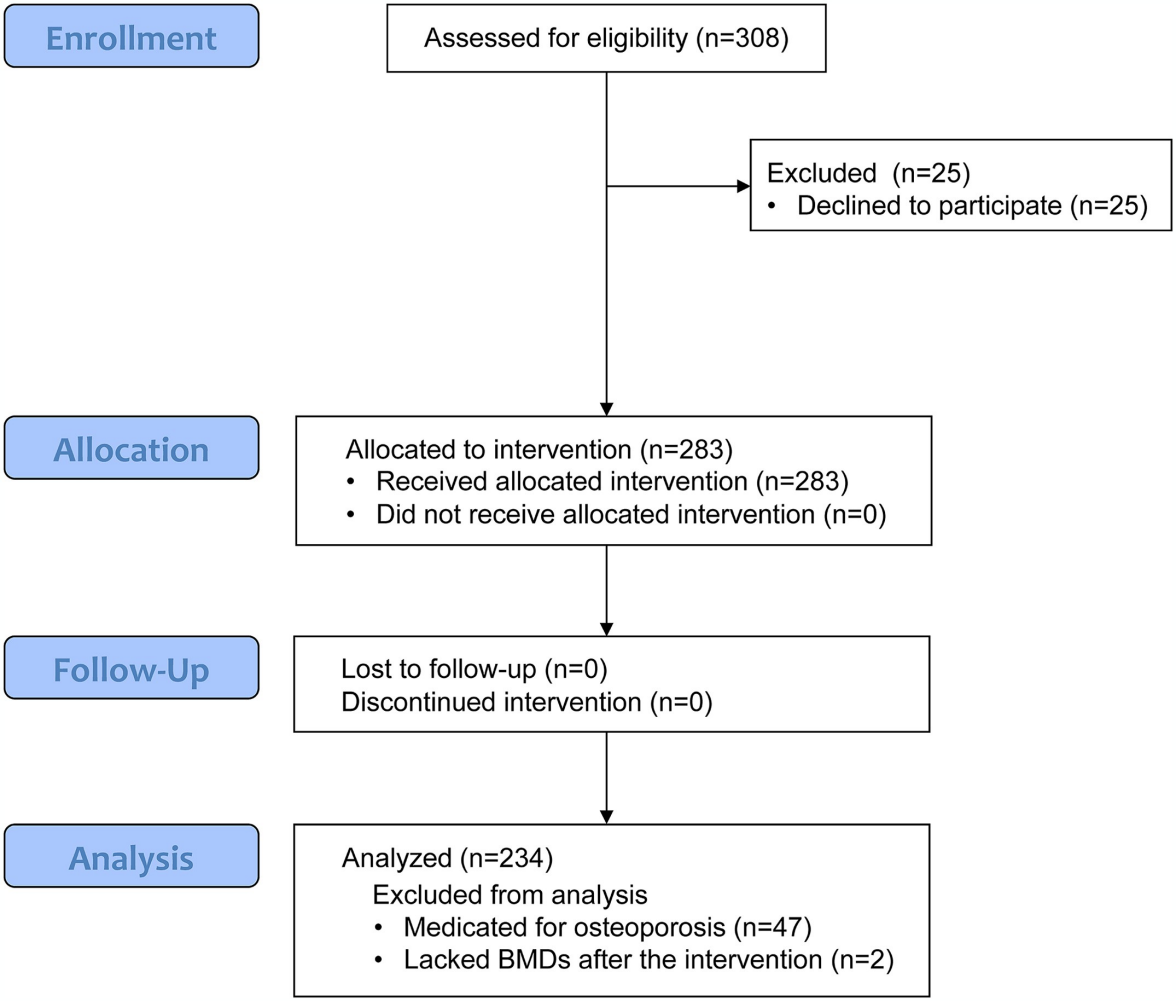
Flow chart of the study. BMD, bone mineral density.

This study is a single-arm intervention design as shown in Fig 1. The protocol for this study and supporting TREND checklist are available as supporting information; see S1 Checklist and S1 and S2 Protocols.

### Subjects and protocol

The study protocol, approved by the Institutional Review Board on Human Experiments, Shinshu University School of Medicine called “I-Rinri-Iinkai” in Japanese (approval no. 639), conformed to the standards set by the Declaration of Helsinki. The study approval was registered in the University hospital Medical Information Network (registration no. UMIN000047428). The registration was completed after subject recruitment because it was not required at the time when the study was conducted. All the subjects provided written informed consent before participating in this study.

We recruited the subjects from postmenopausal women aged ~64 yr who had being participated in the "Jukunen Taiikudaigaku" project for middle-aged and older people in Matsumoto City and performed IWT for ≥6 months. For the recruitment, we distributed leaflets showing the outline of the present study to the participants from September 1^st^ to 15^th^, 2005. Through the recruitment, 283 of 308 candidates provided written informed consent and enrolled in the study (Fig 1). Thereafter, we instructed them to continue IWT as before during the exercise intervention period for the present study from October 16^th^, 2005 to March 15^th^, 2006 while we measured exercise intensity and volume (time) during IWT using a portable calorimeter (the details are shown below). Also, we instructed them to keep their daily activity during the intervention as before. The individual subjects were scheduled to visit Shinshu University Hospital during the periods from October 1^st^ to 15^th^, 2005, and from March 16^th^ to 31^st^, 2006, for BMD measurements before and after the intervention, respectively. The measurements were performed on the second through fourth vertebrae (L2-L4) of LS and both sides of the FN and TH, respectively, by dual-energy X-ray absorptiometry (DXA).

Additionally, to confirm that V̇O_2peak_, isometric knee extension and flexion forces (F_EXT_ and F_FLX_, respectively), and lifestyle-related disease score (LSD score, see the details below) were not changed so much as to influence bone formation during the intervention, we measured their variables during the periods from September 16^th^ to 30^th^, 2005 (Tables 1 and 2) and from March 16^th^ to 31^st^, 2006 (S1 Table) before and after the intervention, respectively. Similarly, to confirm minimal influence of nutritional intake on Ca metabolism, we measured serum concentrations of parathyroid hormone (PTH), calcitonin, and 25-hydroxyvitamin D [25(OH)D] before and after the intervention in 65 of 234 subjects (S2 Table).

**Table 1:** Baseline physical characteristics of subjects for identifying independent factors to increase bone mineral densities by IWT.

Variable	All (n = 234)	LS		FN	
		Lower (n = 64)	Higher (n = 170)	Lower (n = 80)	Higher (n = 154)
Age, yr	64±5 (54–82)	66±5* (55–77)	64±5 (54–82)	67±6*** (56–82)	63±5 (54–74)
Onset of menopause, yr	14±7 (2–37)	16±7 (6–36)	14±8(2–37)	18±8*** (6–37)	12±6 (2–28)
Height, cm	153±5	153±5	153±5	152±5	153.±5
Body mass, kg	55.0±7.8	52.1±6.5***	56.1±8.0	52.0±7.0***	56.5±7.8
BMI, kg/m^2^	23.6±3.3	22.4±3.0**	24.0±3.3	22.5±2.9***	24.1±3.4
% Body fat, %	30.7±6.6	28.8±6.1**	31.5±6.6	28.8±6.3**	31.7±6.5
LS BMD, g/cm^2^	1.027±0.162	0.843±0.070***	1.096±0.130	NA	NA
T-score	0.13±1.36	-1.41±0.59***	0.71±1.09	NA	NA
FN BMD, g/cm^2^	0.789±0.117	NA	NA	0.676±0.053***	0.848±0.097
T-score	0.03±1.06	NA	NA	-1.02±0.48***	0.56±0.89
TH BMD, g/cm^2^	0.864±0.124	NA	NA	NA	NA
T-score	0.01±1.13	NA	NA	NA	NA
V̇O_2peak_, L/min	1.29±0.29	1.24±0.26	1.31±0.30	1.20±0.24***	1.34±0.30
HR_peak_, beats/min	138±14	138±13	138±15	136±14	138±15
*F*_EXT_, Nm	107.8±29.1	105.0±26.5	108.8±30.0	100.5±28.2**	111.5±28.9
*F*_FLX_, Nm	59.7±14.5	58.2±14.3	60.2±14.6	57.6±13.6	60.7±14.9
LSD score	1.5±1.1	1.4±1.0	1.6±1.1	1.3±1.0*	1.7±1.1
Physical activity	1.96±0.62	1.87±0.57	2.00±0.63	1.88±0.57	2.01±0.63

Values are mean ± SD. IWT, interval walking training; LS, lumbar spine; FN, femoral neck; TH, total hip; Lower, subjects with lower baseline bone mineral density (BMD); Higher, subjects with higher baseline BMD; BMI, body mass index; V̇O_2peak_, peak aerobic capacity for walking; HR_peak_, peak heart rate; *F*_EXT_, isometric knee extension force; *F*_FLX_, isometric knee flexion force; LSD score, lifestyle-related disease score; NA, not applicable. Significant differences from the higher group,

* P<0.05,

** P<0.01, and

*** P<0.001.

**Table 2:** Health status of subjects for identifying independent factors to increase BMDs by IWT.

Variable	All (n = 234)	LS		FN	
		Lower (n = 64)	Higher (n = 170)	Lower (n = 80)	Higher (n = 154)
Current alcohol consumers, %	14.1	9.4	15.9	10.0	16.2
Current smokers, %	1.3	1.6	1.2	2.5	0.6
Anamnesis, %					
Hypertension	25.2	26.6	24.7	22.5	26.6
Hyperlipidemia	15.8	15.6	15.9	17.5	14.9
Diabetes mellitus	4.3	3.1	4.7	3.8	4.5
Obesity	25.6	14.1*	30.0	15.0**	31.2

Values are % number of subjects. BMD, bone mineral density; IWT, interval walking training; LS, lumbar spine; FN, femoral neck; Lower, subjects with lower baseline BMD; Higher, subjects with higher baseline BMD. Significant differences from the higher group,

* P<0.05 and

** P<0.01.

### Interval walking training

The IWT in the present study consisted of ≥5 sets of 3-min fast walking at ≥70% of individual pre-training V̇O_2peak_ followed by 3-min slow walking at ~40% V̇O_2peak_ per day, 4 days/week, for 5 months. The reason for adopting this specific protocol was based on the findings from our preliminary study that improvements in physical fitness and lifestyle-related disease symptoms reached plateau levels even when subjects performed fast walking ≥60 min/week. This was later confirmed and published [19]. Training intensity was monitored using a portable calorimeter equipped with a triaxial accelerometer and barometer (JD Mate; Kissei Comtec, Matsumoto, Japan) carried on the midclavicular line of the right or left waist [10, 20]. The device alerted the participants with a beeping signal once per 3 minutes to change the intensity, and a melody informed them every minute when their fast-walking intensity reached the target level. The participants visited a nearby community office every two weeks to transfer their walking records stored in the device to the server computer at Shinshu University via the internet, and they received a report of their training achievements analyzed automatically by the server computer. The report included individual daily walking intensity, energy expenditure, and other training achievement parameters, as shown in Table 3. Based on this report, instructors evaluated how well the participants achieved their target intensity and volume for fast walking, and for participants who were unable to achieve their targets, the instructors encouraged them to achieve the targets accordingly.

**Table 3:** Training achievements for 5 months for identifying independent factors to increase BMDs by IWT.

Variable	All (n = 234)	LS		FN	
		Lower (n = 64)	Higher (n = 170)	Lower (n = 80)	Higher (n = 154)
Walking days per week	2.9±0.1	3.1±0.2	2.9±0.1	2.8±0.2	3.0±0.1
Fast walking					
Time, min/walking day	18±1	19±1	18±1	18±1	18±1
^‡^Energy expenditure, mlO_2_/kg per walking day	287±11	294±20	284±13	273±19	294±14
^‡^Intensity, mlO_2_/kg per minute	15.7±0.2	15.6±0.4	15.8±0.3	15.4±0.4	15.9±0.3
Slow walking					
Time, min/walking day	30±1	28±2	30±1	32±2	29±1
^‡^Energy expenditure, mlO_2_/kg per walking day	242±7	238±12	243±9	240±11	242±10
^‡^Intensity, mlO_2_/kg per minute	8.4±0.2	8.6±0.3	8.4±0.2	8.0±0.3	8.7±0.2
Total fast walking time, min/week	60±3	63±6	59±4	56±5	62±4

Values are mean ± SE. BMD, bone mineral density; IWT, interval walking training; LS, lumbar spine; FN, femoral neck; Lower, subjects with lower baseline BMD; Higher, subjects with higher baseline BMD.

^‡^ Resting oxygen consumption is not included.

### Measurements

#### Exercise intensity estimated by the calorimeter

The calorimeter was equipped with tri-axial accelerometers, as reported in the details of our previous study [20]. Briefly, the oxygen consumption rate (V̇O_2_, ml/min) during walking was calculated according to the equation of V̇O_2_ = 0.056 I_total_ + 249 (r = 0.958, P<0.0001), where I_total_ (N·min) was total impulse and the constant term was V̇O_2_ at rest. The I_total_ was calculated as a product of body mass and a norm of absolute tri-axial accelerations sampled at 10-ms intervals and recorded at every min as an average value. The 95% confidence limit of the equation to estimate V̇O_2_ was ± 90 ml/min over the range of 200–2500 ml/min. Thus, the measurement of V̇O_2_ was accurate enough to estimate the aerobic exercise intensity during IWT in the present study. In addition, since I_total_ represents the average force for a given period to drive the body forward during walking, we thought that the aerobic exercise intensity was proportional to the magnitude of mechanical strain on the bones during IWT and determined it after excluding V̇O_2_ at rest in the present study.

#### V̇O_2peak_

We determined V̇O_2peak_ by measuring energy expenditure with the portable calorimeter and recording at every 5 sec during graded walking on a flat floor at three different speeds—subjectively slow and moderate speeds, as well as subjects’ fastest possible speed—for 3 minutes each, while heart rate (HR) was measured with a near infrared ear pickup probe [10, 21]. The data from the portable calorimeter during the test were converted to V̇O_2_ according to the equation stated above [20]. V̇O_2peak_ and peak HR were determined as their average values for the last 30-second period at the fastest speed during the test, respectively [10, 21]. We confirmed that V̇O_2peak_ measured by the protocol was identical to that by respiratory gas analysis during graded cycling exercise in individuals [10].

#### BMD

The BMDs (g/cm^2^) for LS (L2-L4), FN, and TH were measured by DXA using a Hologic instrument (QDR 4500; Toyo Medic, Tokyo, Japan). The coefficient of variation for BMD measurements was <1.0% for both bone sites [22]. The quality control was conducted before BMD measurement on a daily basis using a proper phantom. The BMDs in both sides of FN and TH were averaged, respectively, for the following analyses.

#### Body mass index (BMI) and %body fat

BMI was calculated as the body mass in kilograms divided by the height in meters squared. Body fat percentage was measured by the impedance method (BF-800; Tanita, Tokyo, Japan).

#### Thigh muscle strength

*F*_EXT_ and *F*_FLX_ in the dominant side of the leg were measured with an isometric force meter (GT330; OG Giken, Okayama, Japan) twice, and the higher value was chosen for analysis.

#### Survey of health status and physical activity

The subjects were interviewed by medical staff and were then asked to fill out questionnaires based on their medical history and their baseline physical activity (Table 1, *bottom*). Physical activity level was scored as 1 = light, 2 = moderate, or 3 = high, according to physical activity and energy requirement guidelines [23].

#### Adherence rate to IWT

The adherence rates to IWT, calculated from total fast walking time per week / target total fast walking per week (60 min) x 100%.

#### Lifestyle-related disease symptoms

To quantify the prevalence of lifestyle-related disease symptoms which has been reported to influence BMDs [15, 16], we calculated the LSD score with reference to Japanese [24] and US [25] healthcare guidelines, as previously described [26]. We added one point when a value met one of the 4 criteria, as follows: 1) BMI ≥25 kg/m^2^; 2) SBP ≥130 mmHg or DBP ≥85 mmHg; 3) triglycerides ≥150 mg/dl, high-density lipoprotein cholesterol <40 mg/dl or low-density lipoprotein cholesterol ≥130 mg/dl; and 4) blood glucose ≥110 mg/dl. Therefore, the maximum total score was 4 points when all criteria were met.

#### Markers for calcium metabolism

To examine any influence of calcium or vitamin D intake during the intervention on BMDs, we determined serum concentrations of high-sensitivity PTH, calcitonin, and 25(OH)D by double-antibody radioimmunoassay (RIA2 antibody assay).

### Analyses and statistics

#### Sample size

This was an exploratory study and the data from previous studies were insufficient; therefore, we did not calculate the sample size setting by power analysis before starting the study.

#### Subjects for the analyses

The data were anonymized and then accessed for research purposes from April 8^th^, 2006. We excluded 47 subjects who visited hospitals or clinics for treatment of osteopenia or osteoporosis by medication after the first BMD measurement (Fig 1). Two of the 236 subjects who performed IWT lacked the BMD measurement after the intervention. Therefore, we analyzed the effects of the training on BMDs in the remaining 234 subjects who declared no treatment and no supplements during the intervention which might have influenced the results of the present study.

#### Identification of independent factors to increase BMDs

Analyses were performed using IBM SPSS Statistics 28 (Armonk, NY). To identify factors independently associated with changes in BMD after the intervention (ΔBMD), we performed multiple regression analyses for each bone site. For the first step of the analyses, we entered into the stepwise model the variables considered as possible independent determinants, which were all baseline physical characteristics, LSD score, and physical activity (Table 1) [7, 12–17], as well as exercise time (in min/week) and intensity (in % individual V̇O_2peak_ before the intervention) at fast and slow walking, respectively (Table 3) [8, 9]. Moreover, although we expected minimal change in V̇O_2peak_, muscle strength, and LSD score after the intervention, we entered all changes after the intervention (S1 Table) into the model. Additionally, we entered alcohol intake, smoking, and health status (Table 2) [14] into the model as dichotomous variables (applicable = 1, not applicable = 0) for each subject. As a result, candidate determinants identified by the analyses were the baseline BMDs for all bone sites, as well as the change in muscle strength for LS, and age and BMI for TH. Then, as the final step of the analyses, we entered these variables into the forced entry model. Additionally, the variables previously reported to influence BMD levels (physical activity, alcohol intake, and smoking) [12–14] were retained in the model (Table 4).

**Table 4:** The results of multiple regression analysis for identifying independent factors to increase BMDs by IWT.

Variable	ΔLS			ΔFN			ΔTH		
	β (95% CI)	Std β	*P* value	β (95% CI)	Std β	*P* value	β (95% CI)	Std β	*P* value
Age, yr	-1.9 x 10^−4^ (-8.8 x 10^−4^ to 5.0 x 10^−4^)	-0.036	0.59	-1.8 x 10^−4^ (-6.3 x 10^−4^ to 2.7 x 10^−4^)	-0.058	0.42	-4.9 x 10^−4^ (-9.0 x 10^−4^ to -0.84 x 10^−4^)	**-0.16**	**0.018**
BMI, kg/m^2^	8.7 x 10^−4^ (-2.8 x 10^−4^ to 20 x 10^−4^)	0.10	0.14	2.4 x 10^−4^ (-4.8 x 10^−4^ to 9.6 x 10^−4^)	0.047	0.51	9.9 x 10^−4^ (3.1 x 10^−4^ to 17 x 10^−4^)	**0.20**	**0.005**
Baseline BMD, g/cm^2^									
for LS	-0.042 (-0.066 to -0.018)	**-0.24**	**0.001**	NA	NA	NA	NA	NA	NA
for FN	NA	NA	NA	-0.027 (-0.049 to -0.0050)	**-0.18**	**0.016**	NA	NA	NA
for TH	NA	NA	NA	NA	NA	NA	-0.042 (-0.062 to -0.023)	**-0.32**	**<0.001**
Δ*F*_EXT_, Nm	-1.4 x 10^−4^ (-3.8 x 10^−4^ to 0.96 x 10^−4^)	0.079	0.24	0.29 x 10^−4^ (-1.2 x 10^−4^ to 1.8 x 10^−4^)	0.027	0.70	0.74 x 10^−4^ (-0.60 x 10^−4^ to 2.1 x 10^−4^)	0.073	0.28
Δ*F*_FLX_, Nm	3.2 x 10^−4^ (-0.31 x 10^−4^ to 6.7 x 10^−4^)	0.12	0.074	-0.25 x 10^−4^ (-2.4 x 10^−4^ to 1.9 x 10^−4^)	-0.016	0.82	0.63 x 10^−4^ (-1.4 x 10^−4^ to 2.6 x 10^−4^)	0.043	0.53
Physical activity	-0.19 x 10^−3^ (-6.2 x 10^−3^ to 5.9 x 10^−3^)	0.0041	0.95	2.1 x 10^−3^ (-1.7 x 10^−3^ to 5.8 x 10^−3^)	0.075	0.27	1.6 x 10^−3^ (-1.9 x 10^−3^ to 5.0 x 10^−3^)	0.059	0.38
Current alcohol consumers	-5.9 x 10^−3^ (-17 x 10^−3^ to 5.0 x 10^−3^)	-0.071	0.29	0.43 x 10^−3^ (-6.2 x 10^−3^ to 7.1 x 10^−3^)	0.087	0.90	1.4 x 10^−3^ (-4.7 x 10^−3^ to 7.5 x 10^−3^)	0.030	0.65
Current smokers	8.7 x 10^−3^ (-25 x 10^−3^ to 42 x 10^−3^)	0.034	0.61	-0.84 x 10^−3^ (-21 x 10^−3^ to 20 x 10^−3^)	-0.0055	0.94	-7.3 x 10^−3^ (-26 x 10^−3^ to 12 x 10^−3^)	-0.050	0.45

BMD, bone mineral density; IWT, interval walking training; Δ, the change after the exercise intervention; LS, lumbar spine; FN, femoral neck; TH, total hip; BMI, body mass index; *F*_EXT_, isometric knee extension force; *F*_FLX_, isometric knee flexion force; β, unstandardized coefficient; Std β, standardized coefficient; NA, not applicable. Values in boldface indicate significant determinants. The analysis was performed on all subjects (n = 234).

#### Analyses of the relationships between the baseline BMDs vs ΔBMDs

**Fig 2:**
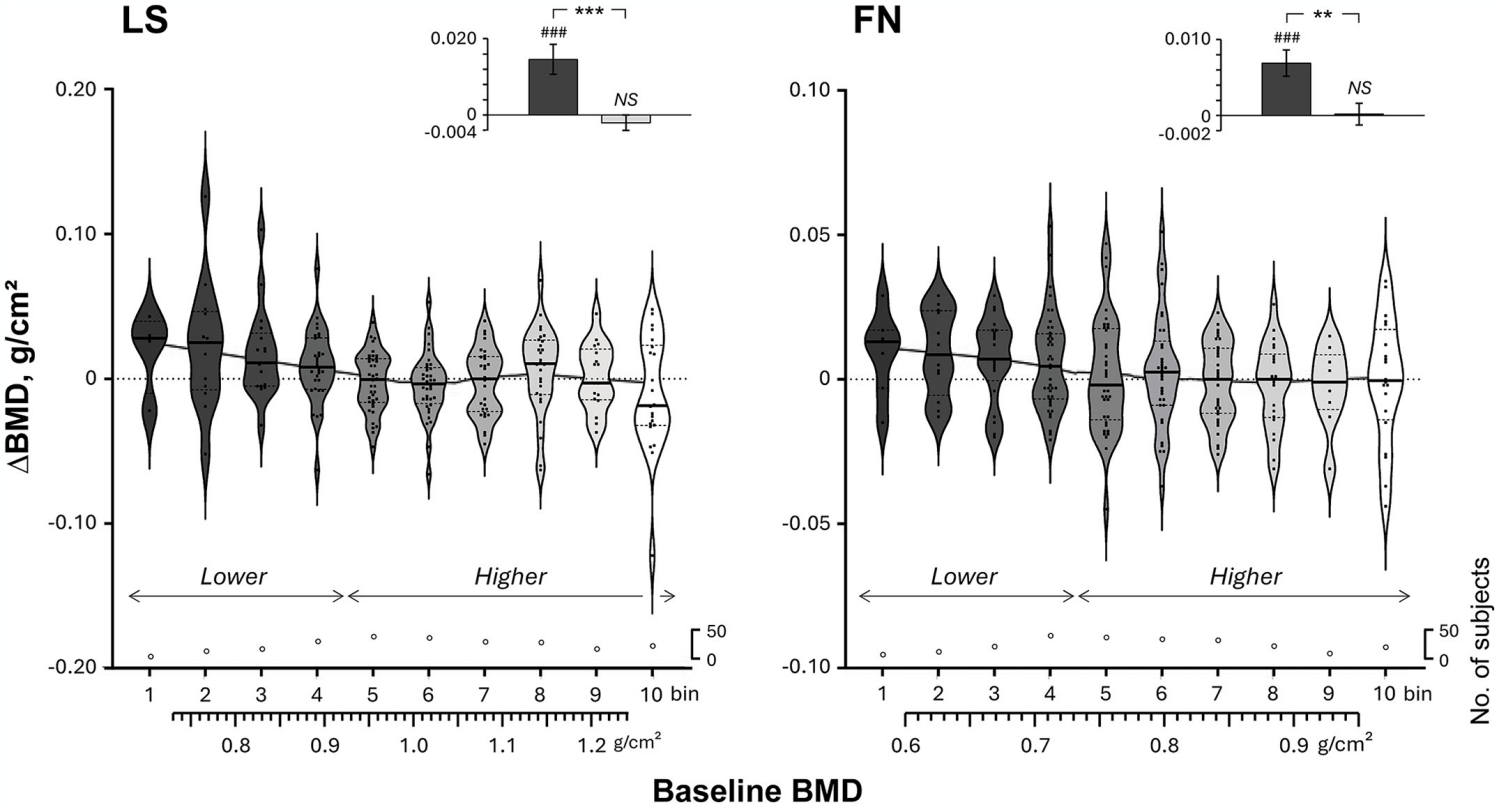
Individual baseline vs change (Δ) in bone mineral density (BMD) values for the lumbar spine (LS) and bilateral femoral neck (FN) after the exercise intervention using violin plots, together with the locally weighted scatterplot smoothing (LOWESS) trendline. The baseline BMDs and the corresponding ΔBMDs were divided into 10 bins: <0.735 g/cm^2^, a 0.063-g/cm^2^ increment from 0.735 to 1.239 g/cm^2^, and ≥1.239 g/cm^2^ for LS; and <0.605 g/cm^2^, a 0.043-g/cm^2^ increment from 0.605 to 0.949 g/cm^2^, and ≥0.949 g/cm^2^ for FN. In the violin plots, thick lines indicate median and dotted lines indicate 25% and 75% quartiles for each bin. The number of subjects included in each bin was also presented in the bottom of the panels. The upper panels show the means and SE bars of ΔBMD after the intervention in the lower and higher groups for LS and FN, respectively, where subjects in the lower 4 bins and the higher 6 bins of the baseline BMD were pooled, respectively. ^###^Significant differences from the baseline, P<0.001. Significant differences between the lower and higher groups, **P<0.01 and ***P<0.001.

For any bone sites where the multiple regression analyses above identified the baseline BMDs as only significant determinants for increasing BMDs (this was the case for LS and FN; see the Results section for further details) (Table 4), we performed the following analyses. First, using violin plots we depicted individual ΔBMDs against the baseline BMDs, divided into 10 bins, to assess both interindividual variability and overall trends (main panels of Fig 2, see the figure legend for further details). We then analyzed the relationship between baseline BMDs (*X*) vs ΔBMDs (*Y*) by the locally weighted scatterplot smoothing (LOWESS) method [27].

According to the relationship between baseline BMDs vs ΔBMDs (see the Results section for further details), we divided subjects into lower and higher BMD groups and compared ΔBMD between the groups for each bone site (upper panels of Fig 2).

To consider any underlying mechanisms for the different responses to IWT between the lower and higher BMD groups, we compared the average values of baseline physical characteristics (Table 1), health status (Table 2), training achievements (Table 3), and their changes after the intervention (S1 Table) between the lower and higher BMD groups for LS and FN, respectively.

#### Statistics for comparisons between groups

One-way analysis of variance (ANOVA) was used to examine any significant differences in baseline physical characteristics, physical activity (Table 1), training achievements (Table 3), and serum concentrations of PTH, calcitonin, and 25(OH)D (S2 Table) between the lower and higher BMD groups for LS and FN. This model was also used to examine any significant differences in changes in physical fitness and LSD score after the intervention between the groups (S1 Table). Fisher’s exact probability test was used to examine any significant frequency differences in dichotomous variables between the lower and higher BMD groups for LS and FN (Table 2). Two-way [groups (lower vs higher BMD) x time (before vs after the intervention)] ANOVA for repeated measures was used to examine any significant differences in the effects of training on BMDs for LS and FN (Fig 2 upper panels). This model was also used to examine any significant changes in serum concentrations of PTH, calcitonin, and 25(OH)D after the intervention between the lower and higher BMD groups for LS and FN (S2 Table). Analysis of covariance (ANCOVA) was used to examine any significant differences in ΔBMDs between the lower and higher BMD groups for LS and FN, respectively, with other variables (Tables 1 and 3 and S1 Table) included as covariates. All P values <0.05 were considered significant. Values are presented as mean ± standard errors (SE) except for Table 1 and the main panels of Fig 2.

## Results

### Identification of independent factors to increase BMDs

Table 4 shows the results of the final model of the multiple regression analyses. We found that significant independent determinants for ΔBMDs were only the baseline BMDs for LS and FN (both, P<0.02), whereas they were age and BMI as well as the baseline BMD for TH (all, P<0.02). Other items of baseline physical characteristics—including anthropological variables, V̇O_2peak_, thigh muscle strength, physical activity (Table 1), as well as health status including current alcohol consumption and smoking, plus lifestyle-related disease symptoms (Table 2)—were not significant determinants of ΔBMDs for any bone sites (all, P>0.05).

We also found that the training achievements—exercise intensity for fast walking and total fast walking time (Table 3)—were not significant determinants of ΔBMDs for any bone sites (all, P>0.05).

Furthermore, we found that the changes in physical fitness and LSD scores during training (S1 Table) were not significant determinants of ΔBMDs for any bone sites (all, P>0.07).

### Analyses for the relationship between the baseline BMDs vs ΔBMDs

Accordingly, we examined how the baseline BMDs affected ΔBMDs for LS and FN. Fig 2 presents violin plots depicting baseline values vs ΔBMDs for LS and FN after the intervention, together with the LOWESS trendline. As shown in the figure, the median values of ΔBMD were consecutively higher than zero in the lower 4 bins while not in the remaining higher 6 bins for both LS and FN. Also, the LOWESS trendline shows that ΔBMD responses were attenuated as the baseline BMDs increased in the lower 4 bins and then showed a flat line (Y = ~0) in the higher 6 bins. Accordingly, we divided subjects into the lower 4 bins (lower BMD group) and the higher 6 bins (higher BMD group) for each bone site.

The upper panels of Fig 2 show ΔBMDs after the intervention for LS and FN. BMDs for LS and FN increased significantly in the lower BMD groups, respectively (both, P<0.001), while they did not change significantly in the higher BMD groups, respectively (both, P>0.3). We found significantly greater ΔBMDs in the lower than in the higher BMD groups (both, P<0.01). The increases in the lower groups were equivalent to 1.8% and 1.0% of the baseline values for LS and FN, respectively.

We showed the baseline physical characteristics (Table 1) and health status of subjects (Table 2), their training achievements completed by the 234 subjects over 5 months (Table 3), and the effects of training (S1 Table) in the lower and higher BMD groups for LS and FN, respectively.

As shown in Table 1, the lower BMD groups for LS and FN were significantly older, and their body mass, BMI, %body fat, mean BMD, and T score values were significantly lower than those in the higher BMD groups (all, P<0.05). The lower BMD group for FN also had significantly lower V̇O_2peak_, *F*_EXT_, and LSD score values than those in the higher BMD group (all, P<0.05). However, we confirmed that no items of these physical characteristics had a significant effect on ΔBMDs in the comparison between the lower and higher BMD groups by ANCOVA (all, P>0.13), consistent with the results that baseline BMDs were the major determinants of ΔBMDs for LS and FN by the multiple regression analyses.

On the other hand, as shown in Table 2, we found no significant differences in any items of health status between the lower and higher BMD groups in either bone site (all, P>0.23) except for a significantly lower prevalence of obesity in the lower than the higher group for both bone sites (both, P<0.02). Similarly, as shown in Table 3, we found no significant differences in training achievements between the groups for either bone site (all, P>0.05). Furthermore, after the intervention, we found no significant differences in the changes in any items between the groups (all, P>0.14) (S1 Table). Finally, we found no significant differences in the baselines of serum concentrations of PTH, calcitonin, and 25(OH)D before the intervention and their changes after the intervention between the groups in either bone site except for slightly but significantly lower 25(OH)D in the lower BMD group than in the higher BMD group for each bone site (both, P<0.05) (S2 Table), consistent with previous observations [28].

### Adverse events

The subjects completed a questionnaire regarding the presence and degree of chronic joint pain after the intervention. Ten subjects (3.5%) complained of mild exacerbation of joint pain after the intervention whereas no other adverse event was observed.

## Discussion

In the present study, we found that significant determinants for increasing BMDs by IWT were the baseline BMDs for all bone sites, as well as age and BMI for TH. Moreover, the relationships between the baseline BMDs vs ΔBMDs for LS and FN showed that subjects with lower baseline BMD had obvious increases in BMD after the intervention while those with higher baseline BMD did not. Accordingly, we considered any underlying mechanisms for the different responses. These findings were obtained along with minimal adverse events.

### The baseline physical characteristics and activity of subjects

Table 1 shows the baseline physical characteristics and health status of the subjects. The average body mass, BMI, and BMDs for LS, FN, and TH in all subjects were approximately equal to the values of Japanese women aged 60–69 years reported by previous studies [29]. In addition, the average V̇O_2peak_, *F*_EXT_, and *F*_FLX_ belonged to the middle physical fitness level of Japanese women of the same age [30]. Similarly, the health status of the subjects (Table 2) was almost identical to that of the age-matched Japanese women [31]. Thus, the subjects in the present study reflected the physical characteristic variation of “moderately active” postmenopausal women of the same age in Japan with age-associated declines of body mass, physical fitness, and BMDs as suggested in the previous studies [2, 31–34].

### High training achievements

As shown in Table 3, the subjects accomplished IWT by ~80% of V̇O_2peak_ of fast walking and by the adherence rate of ~100%, on average, during the 5 months of training.

The intensity of fast walking accomplished by the subjects was very high and close to the maximal range of exercise intensity recommended by ACSM for ensuring safety [35]. This might be enabled by the unique exercise style of IWT. Generally speaking, many older people unaccustomed to regular exercise do not like to perform fast walking because of the breathlessness, tachycardia, and muscle soreness they often feel shortly after undertaking high-intensity exercise. However, with IWT they usually find they can persevere in fast walking, if only for short time, and when they are unable to continue, they are allowed to slow down. After the brief respite provided by slow walking, during which the feeling of fatigue is reduced, they once again feel motivated to perform fast walking [10]. Thus, during the training program for 5 months, the subjects in the present study accomplished fast walking at 20–40% higher intensity than the values recommended by the ACSM [4] (Table 3).

### The identification of independent factors to increase BMDs by IWT

Using multiple regression analyses, we found that the baseline BMDs were significant independent determinants for increasing BMDs for LS and FN, whereas other items of baseline physical characteristics and health status were not (Table 4).

There were several cross-sectional studies showing that some items of physical characteristics and health status influence BMDs [14, 17]. In the present study, to confirm the factors influencing baseline BMDs, we performed additional multiple regression analyses (S1 File) and found that these items were significant independent determinants for the “baseline” BMDs (S3 Table) but not for ΔBMDs for LS and FN. In other words, since the baseline BMDs included the effects of these variables on ΔBMDs, they were not detected by the multiple regression analyses.

On the other hand, we found the age and BMI as well as the baseline BMD were significant independent determinants for increasing BMD for TH. This might be because the increasing BMD effect by physical exercise on BMDs was reportedly much less for TH than for LS and FN [36] so that items of physical characteristics were detected.

### The relationships between the baseline BMDs vs ΔBMDs and the underlying mechanisms

As shown in Fig 2, we found that BMDs for LS and FN increased after the intervention, but the increases (ΔBMDs) were attenuated as the baseline BMDs increased. After dividing subjects into the lower and higher BMD groups, we considered any underlying mechanisms for the different response to IWT between the groups.

The significance of aerobic exercise intensity relative to individual V̇O_2peak_ during aerobic exercise for increasing BMDs in postmenopausal women has been broadly accepted [4]. For example, Borer et al. [8] examined the effects of exercise intensity for aerobic training on BMD. They divided 25 postmenopausal women into 2 groups: walking at low and high intensities, equivalent to 67% and 86% baseline V̇O_2peak_, respectively, at a commercial mall, 4.8 km a day, 4 days a week, for 15 weeks, and found that BMDs of legs and total body, but not in other sites, were preserved in the subjects in the high-intensity group while decreased by ~1.5% in the low-intensity group with the critical exercise intensity for the preservation at 74% of V̇O_2peak_ [8]. Similarly, Hatori et al. [37] divided 33 postmenopausal women into 3 groups: a sedentary group and 2 groups walking on a treadmill in a gym at below and above individual aerobic thresholds, respectively, 30 min a day, 3 days a week, for 7 months, where the exercise intensity during the training was confirmed from heart rate monitored by ambulatory electrocardiography. They found that BMD in the lumbar vertebrae decreased by 1.7% and 1.0% in the sedentary and low-intensity groups, respectively, while it increased by 1.1% in the high-intensity group.

On the other hand, to our knowledge, very few studies have carefully examined the influence of the baseline BMDs on the effects of aerobic exercise on BMDs. In fact, the only paper we could find was that by Winters-Stone and Snow [7]. They had subjects, who were premenopausal and ~25 yr younger than the subjects in the present study, undergo 100 jumps and 100 repetitions of lower-body resistance exercise (squat, lunges, and calf raises) per day, 3 days a week, for a year. The training program per day consisted of 9 sets of 10–12 jumps intermitted with 15–30 sec of rest and 9 sets of lower-body resistance exercises intermitted with 2–3 min rest. They found that % change in trochanter BMD after the training was inversely correlated with their baseline values; ~5% increase in subjects with 0.5 g/cm^2^ and ~0% increase in those with 0.9 g/cm^2^ of the BMD. These collective results from the previous studies on the influence of the baseline BMDs [7] and exercise intensity for increasing BMDs [8, 37] were consistent with the results in the present study that exercise training at intensity of ≥70% V̇O_2peak_ increased BMDs, which had greater effects on subjects with lower rather than higher baseline BMDs.

These results might be explained by the dissociation between the indices for aerobic stress to cardio-respiratory function and for the magnitude of mechanical strain on the bones during aerobic exercise.

When we compared the average baseline BMDs and V̇O_2peak_ between the lower and higher BMD groups in each bone site (Table 1), the baseline BMDs were 30% and 25% higher in the higher BMD group than those in the lower BMD group for LS and FN, respectively. These results suggest that to increase BMDs for LS and FN in the higher BMD group by a similar degree to the lower BMD group, subjects in the group had needed to perform fast walking at more than 25% higher intensity than in the lower BMD group, assuming that the mechanical strain to the bones required to increase the density was proportional to the baseline BMDs in line with the “mechanostat” theory [11] and that the strain to the bones increased as the aerobic exercise strain increased. On the other hand, as shown in Table 3, aerobic exercise intensity for fast walking during the training was only 1% and 3% higher in the higher BMD group than in lower BMD group for LS and FN, respectively, suggesting that much higher aerobic exercise intensity had been needed to increase BMDs in the higher BMD group.

However, such higher intensity was not possible because exercise intensities during fast walking in the lower and higher BMD groups had reached the maximal range recommended by ACSM for ensuring safety [35]. Thus, other types of exercise training than walking, which do not need so much loading on cardio-respiratory function, such as jumping or resistance training, might be more appropriate for subjects in the higher BMD groups if they want to increase their BMDs, although these types of exercise are likely to be more difficult than walking as activities postmenopausal women would continue as habitual exercise.

### Limitations

Since we did not designate any sedentary control groups in the present study, we could not evaluate effects of IWT alone on BMDs. However, when we assumed that BMDs decreases by ~1% per year (~0.5% per 6 months) on average in sedentary subjects of the same age according to the results of previous studies [2, 37, 38], the BMDs after the intervention in the lower and higher BMD groups would be ~1.9% and ~0.5% higher than the corresponding values in possible sedentary control groups, respectively.

We observed no significant increases in BMDs from the baselines after the intervention in the higher BMD groups (upper panels of Fig 2) due to the insufficient mechanical strain to the bones; however, considering the possible decrease in BMDs with aging for subjects with a sedentary lifestyle, IWT may be of benefit to the groups for maintaining their BMDs.

We could not determine strict cut-off values of the baseline BMDs for increasing BMDs for LS and FN since the changes in BMDs after the intervention were rather varied (main panels of Fig 2). Nevertheless, according to the relationships between the baseline BMDs vs ΔBMDs in the figure, they might be located around the boundary between the 4^th^ and 5^th^ bins for each bone site, which was assumed to be 0.924 g/cm^2^ for LS and 0.734 g/cm^2^ for FN.

While we used the linear term of baseline BMDs directly in the multiple regression analyses to identify significant determinants of ΔBMDs (Table 4), the precise analysis between the baseline BMDs vs ΔBMDs for LS and FN showed that the relationships between them were not linear (main panels of Fig 2). Accordingly, we performed additional multiple regression analyses, where baseline BMDs were entered into the model as dichotomous variables (lower BMD group = 1, higher BMD group = 0), and reconfirmed that the baseline BMDs were only the significant determinants of ΔBMDs for LS and FN (both, P<0.003).

We did not determine calcium to phosphorus ratio (Ca/P) in habitual diets or did not determine other bone turnover markers. Regarding dietary Ca/P, the lower ratio was reported to induce higher serum PTH to increase bone resorption [39]. In the present study, we confirmed that there were no significant differences in serum PTH values before the intervention and their changes after the intervention between the lower and higher BMD groups (S2 Table), suggesting minimal effects of Ca/P on the results, if any, though it was with a limited number of subjects.

We did not perform further analyses on TH because age and BMI as well as baseline BMD were significant determinants of ΔBMD for TH by multiple regression analyses. Moreover, in addition to these direct effects, we also found significant interactive effects of [age x baseline BMD] on ΔBMD for TH (P<0.001). These interactive as well as direct effects made it difficult to analyze the relationship between the baseline BMD vs ΔBMD for TH as we did for LS and FN (Fig 2) after adjustment for these variables as covariates.

### Clinical implication

The T scores of a large portion of the subjects in the present study were higher than the diagnostic criterion for osteopenia of -1.0, while the lower groups for LS and FN could be considered to have osteopenia at that site (Table 1). When we compared the values ± SDs for T scores with those of the BMDs in Table 1, we found that 0.01 g/cm^2^ of BMD was almost equivalent to 0.1 of the T score at both LS and FN. Since BMD in the lower BMD groups increased on average by 0.015 and 0.007 g/cm^2^ for LS and FN, respectively, for the 5-month training (upper panels of Fig 2), and since their T scores before the intervention were -1.41 and -1.02, respectively (Table 1), this finding shows that these subjects had recovered 9% of their bone loss from the young adult mean BMD (YAM, T score = 0.0) for the 5 months of the training. If they were to continue the training thereafter, their BMDs would likely be close to the YAM in several years though the speed would go down as the baseline BMDs increased. Even if their BMDs did not recover to the YAM, we speculate that these individuals could maintain a T score of greater than -1.0 throughout the remainder of their lifetime. Additionally, T scores in the higher groups for LS and FN were 0.71 and 0.56 before the intervention, respectively, higher than the YAM, which were maintained after the intervention. Together, these results suggest that IWT would be beneficial to most postmenopausal women who desire to decrease their risk of experiencing bone fractures.

## Conclusions

We found that significant determinants for increasing BMDs by 5-month IWT were baseline BMDs for all bone sites, as well as age and BMI for TH. Moreover, the relationships between the baseline BMDs vs ΔBMDs for LS and FN showed that IWT may be of benefit, with minimal adverse events, to postmenopausal women although the effects were greater in those with lower baseline BMDs.

## Supporting information

S1 Checklist TREND checklist. (PDF)

S1 Protocol The protocol for the clinical study. (PDF)

S2 Protocol The protocol for the clinical study in the original language (in Japanese). (PDF)

S1 File Supplementary methods and results. (DOCX)

S1 Table Changes in physical fitness and LSD score from baseline after the intervention for identifying independent factors to increase BMDs by IWT. (DOCX)

S2 Table Markers for calcium metabolism before and after the intervention for examining any influence on the differences in ΔBMDs between the lower and higher BMD groups. (DOCX)

S3 Table The results of multiple regression analysis for confirming independent factors influencing the baseline BMDs before the intervention. (DOCX)

## Abbreviations

25(OH)D: 25-hydroxyvitamin D
BMD: bone mineral density
BMI: body mass index
DXA: dual-energy X-ray absorptiometry
F_EXT_ and F_FLX_: isometric knee extension and flexion forces, respectively
FN: femoral neck
IoT: Internet of Things
IWT: interval walking training
LS: lumbar spine
LSD score: lifestyle-related disease score
PTH: parathyroid hormone
TH: total hip
V̇O_2peak_: peak aerobic capacity
YAM: young adult mean BMD

## References

[1] Cummings SR, Melton LJ. Epidemiology and outcomes of osteoporotic fractures. Lancet. (2002) 359:1761–7. doi: 10.1016/S0140-6736(02)08657-9 12049882

[2] Nguyen TV, Sambrook PN, Eisman JA. Bone loss, physical activity, and weight change in elderly women: the Dubbo Osteoporosis Epidemiology Study. J Bone Miner Res. (1998) 13:1458–67. doi: 10.1359/jbmr.1998.13.9.1458 9738519

[3] Bliuc D, Alarkawi D, Nguyen TV, Eisman JA, Center JR. Risk of subsequent fractures and mortality in elderly women and men with fragility fractures with and without osteoporotic bone density: the Dubbo Osteoporosis Epidemiology Study. J Bone Miner Res. (2015) 30:637–46. doi: 10.1002/jbmr.2393 25359586

[4] American College of Sports Medicine. “Exercise prescription for populations with other chronic diseases and health conditions”. In: Riebe D, editor. ACSM’s Guidelines for Exercise Testing and Prescription (9th ed). Baltimore, MD: Lippincott Williams & Wilkins. (2014). p. 260–354.

[5] Voukelatos A, Merom D, Sherrington C, Rissel C, Cumming RG, Lord SR. The impact of a home-based walking programme on falls in older people: the Easy Steps randomised controlled trial. Age Ageing. (2015) 44:377–83. doi: 10.1093/ageing/afu186 25572426

[6] Benedetti MG, Furlini G, Zati A, Letizia Mauro G. The effectiveness of physical exercise on bone density in osteoporotic patients. Biomed Res Int. (2018) 2018:4840531. doi: 10.1155/2018/4840531 30671455 PMC6323511

[7] Winters-Stone KM, Snow CM. Musculoskeletal response to exercise is greatest in women with low initial values. Med Sci Sports Exerc. (2003) 35:1691–6. doi: 10.1249/01.MSS.0000089338.66054.A5 14523306

[8] Borer KT, Fogleman K, Gross M, La New JM, Dengel D. Walking intensity for postmenopausal bone mineral preservation and accrual. Bone. (2007) 41:713–21. doi: 10.1016/j.bone.2007.06.009 17686670

[9] Zheng Q, Kernozek T, Daoud-Gray A, Borer KT. Anabolic bone stimulus requires a pre-exercise meal and 45-minute walking impulse of suprathreshold speed-enhanced momentum to prevent or mitigate postmenopausal osteoporosis within circadian constraints. Nutrients. (2021) 13:3727. doi: 10.3390/nu13113727 34835982 PMC8620686

[10] Masuki S, Morikawa M, Nose H. Internet of things (IoT) system and field sensors for exercise intensity measurements. Compr Physiol. (2020) 10:1207–1240. doi: 10.1002/cphy.c190010 32941686

[11] Frost HM. Bone’s mechanostat: a 2003 update. Anat Rec A Discov Mol Cell Evol Biol. (2003) 275:1081–101. doi: 10.1002/ar.a.10119 14613308

[12] Whalen RT, Carter DR, Steele CR. Influence of physical activity on the regulation of bone density. J Biomech. (1988) 21:825–37. doi: 10.1016/0021-9290(88)90015-2 3225269

[13] Madansingh SI, Ngufor CG, Fortune E. Quality over quantity: skeletal loading intensity plays a key role in understanding the relationship between physical activity and bone density in postmenopausal women. Menopause. (2020) 27:444–449. doi: 10.1097/GME.0000000000001486 31895180 PMC7108051

[14] LeBoff MS, Greenspan SL, Insogna KL, Lewiecki EM, Saag KG, Singer AJ, et al. The clinician’s guide to prevention and treatment of osteoporosis. Osteoporos Int. (2022) 33:2049–2102. doi: 10.1007/s00198-021-05900-y 35478046 PMC9546973

[15] Masugata H, Senda S, Inukai M, Murao K, Hosomi N, Iwado Y, et al. Association between bone mineral density and arterial stiffness in hypertensive patients. Tohoku J Exp Med. (2011) 223:85–90. doi: 10.1620/tjem.223.85 21263208

[16] Sugimoto T, Sato M, Dehle FC, Brnabic AJ, Weston A, Burge R. Lifestyle-related metabolic disorders, osteoporosis, and fracture risk in Asia: A systematic review. Value Health Reg Issues. (2016) 9:49–56. doi: 10.1016/j.vhri.2015.09.005 27881259

[17] Saddik H, Pinti A, Antoun A, Al Rassy N, El Hage Z, Berro AJ, et al. Limb muscular strength and bone mineral density in elderly subjects with low skeletal muscle mass index. J Clin Densitom. (2021) 24:538–547. doi: 10.1016/j.jocd.2021.03.011 33958260

[18] Morikawa M, Masuki S, Furuhata S, Shimodaira H, Furihata M, and Nose H. Interval walking training over 10 years protects against age-associated declines in physical fitness (abstract). FASEB J. (2018). p. 588.9.28939591

[19] Masuki S, Morikawa M, Nose H. High-intensity walking time is a key determinant to increase physical fitness and improve health outcomes after interval walking training in middle-aged and older people. Mayo Clin Proc. (2019) 94:2415–2426. doi: 10.1016/j.mayocp.2019.04.039 31477320

[20] Iwashita S, Takeno Y, Okazaki K, Itoh J, Kamijo Y, Masuki S, et al. Triaxial accelerometry to evaluate walking efficiency in older subjects. Med Sci Sports Exerc. (2003) 35:1766–72. doi: 10.1249/01.MSS.0000089350.54959.CB 14523318

[21] Nemoto K, Gen-no H, Masuki S, Okazaki K, Nose H. Effects of high-intensity interval walking training on physical fitness and blood pressure in middle-aged and older people. Mayo Clin Proc. (2007) 82:803–11. doi: 10.4065/82.7.803 17605959

[22] Hologic. QDR 4500 Acclaim Series catalog. 1999.

[23] Ministry of Health, Labor and Welfare of Japan. “Energy”. In: Dietary Reference Intakes for Japanese 2010. Tokyo: Daiichi Shuppan (2010). p. 43–61.

[24] Definition and the diagnostic standard for metabolic syndrome—Committee to Evaluate Diagnostic Standards for Metabolic Syndrome [in Japanese]. Nihon Naika Gakkai Zasshi. (2005) 94:794–809. 15865013

[25] National Cholesterol Education Program (NCEP) Expert Panel on Detection, Evaluation, and Treatment of High Blood Cholesterol in Adults (Adult Treatment Panel III). Third Report of the National Cholesterol Education Program (NCEP) Expert Panel on Detection, Evaluation, and Treatment of High Blood Cholesterol in Adults (Adult Treatment Panel III) final report. Circulation. (2002) 106:3143–421. 12485966

[26] Masuki S, Mori M, Tabara Y, Sakurai A, Hashimoto S, Morikawa M, et al. The factors affecting adherence to a long-term interval walking training program in middle-aged and older people. J Appl Physiol. (2015) 118:595–603. doi: 10.1152/japplphysiol.00819.2014 25539937

[27] Cleveland WS. Robust Locally Weighted Regression and Smoothing Scatterplots. J Am Stat Assoc. (1979) 74:829–836.

[28] Khaw KT, Sneyd MJ, Compston J. Bone density parathyroid hormone and 25-hydroxyvitamin D concentrations in middle aged women. BMJ. (1992) 305:273–7. doi: 10.1136/bmj.305.6848.273 1392857 PMC1882724

[29] Tokida R, Uehara M, Nakano M, Suzuki T, Sakai N, Ikegami S, et al. Reference values for bone metabolism in a Japanese cohort survey randomly sampled from a basic elderly resident registry. Sci Rep. (2021) 11:7822. doi: 10.1038/s41598-021-87393-7 33837266 PMC8035137

[30] Morikawa M, Okazaki K, Masuki S, Kamijo Y, Yamazaki T, Gen-no H, et al. Physical fitness and indices of lifestyle-related diseases before and after interval walking training in middle-aged and older males and females. Br J Sports Med. (2011) 45:216–24. doi: 10.1136/bjsm.2009.064816 19846423

[31] Amagai Y, Ishikawa S, Gotoh T, Kayaba K, Nakamura Y, Kajii E. Age at menopause and mortality in Japan: the Jichi Medical School Cohort Study. J Epidemiol. (2006) 16:161–6. doi: 10.2188/jea.16.161 16837767 PMC7603913

[32] Kanis JA. Diagnosis of osteoporosis and assessment of fracture risk. Lancet. (2002) 359:1929–36. doi: 10.1016/S0140-6736(02)08761-5 12057569

[33] Lins VieiraNF, da Silva NascimentoJ, do NascimentoCQ, Barros NetoJA, Oliveira Dos SantoACS. Association between bone mineral density and nutritional status, body composition and bone metabolism in older adults. J Nutr Health Aging. (2021) 25:71–76. doi: 10.1007/s12603-020-1452-y 33367465

[34] Kapuš O, Gába A, Lehnert M. Relationships between bone mineral density, body composition, and isokinetic strength in postmenopausal women. Bone Rep. (2020) 12:100255. doi: 10.1016/j.bonr.2020.100255 32181269 PMC7063090

[35] American College of Sports Medicine. “General principles of Exercise prescription”. In: Riebe D, editor. ACSM’s Guidelines for Exercise Testing and Prescription (9th ed). Baltimore, MD: Lippincott Williams & Wilkins. (2014). p. 162–193.

[36] Hejazi K, Askari R, Hofmeister M. Effects of physical exercise on bone mineral density in older postmenopausal women: a systematic review and meta-analysis of randomized controlled trials. Arch Osteoporos. (2022) 17:102. doi: 10.1007/s11657-022-01140-7 35896850

[37] Hatori M, Hasegawa A, Adachi H, Shinozaki A, Hayashi R, Okano H, et al. The effects of walking at the anaerobic threshold level on vertebral bone loss in postmenopausal women. Calcif Tissue Int. (1993) 52:411–4. doi: 10.1007/BF00571327 8369985

[38] Watson SL, Weeks BK, Weis LJ, Harding AT, Horan SA, Beck BR. High-intensity resistance and impact training improves bone mineral density and physical function in postmenopausal women with osteopenia and osteoporosis: the LIFTMOR randomized controlled trial. J Bone Miner Res. (2018) 33:211–220. doi: 10.1002/jbmr.3284 28975661

[39] Kemi VE, Kärkkäinen MUM, Rita HJ, Laaksonen MML, Outila TA, Lamberg-Allardt CJE. Low calcium:phosphorus ratio in habitual diets affects serum parathyroid hormone concentration and calcium metabolism in healthy women with adequate calcium intake. Br J Nutr. (2010) 103:561–568. doi: 10.1017/S0007114509992121 19781123

