# Supplementary material for: Increased response of postmenopausal bone to interval walking training depends on baseline bone mineral density
Source: PLoS One. 2024 Sep 5;19(9):e0309936. doi: 10.1371/journal.pone.0309936 (PMC11376574; doi:10.1371/journal.pone.0309936)
Supplement: S1 Protocol — (PDF) [file pone.0309936.s002.pdf]

Voluntary clinical research

The study on the preventive and therapeutic effects of increased physical fitness by interval walking training on age-associated diseases in middle-aged and older people

Protocol

A single-arm intervention study

Study director

Hiroshi Nose

Professor, Shinshu University Graduate School of Medicine

1. Purpose and significance

Exercise training is one of the most effective strategies for decreasing the likelihood of age- and lifestyle-related diseases, thereby promoting independence and enhancing the quality of life in the rapidly growing elderly populations of many countries (1, 2). Recent guidelines suggest that to achieve the desired effects, an exercise program should be tailored to an individual's fitness level, and in most cases, individualized training is performed using exercise equipment, stationary bicycles, and treadmills at a gymnasium or related facility where the exercise intensity can be more easily monitored. However, these training programs are costly and limit adherence to training programs (3).

To solve these problems, we recently developed a broadly available, remotely supervised exercise training system for middle-aged and older individuals. The system consists of interval walking training (IWT) and an IT network for monitoring exercise intensity and volume during IWT for a large population of middle-aged and older people. The IWT is a training regimen repeating ≥5 sets of fast walking at ≥70% individual peak aerobic capacity (VO_{2peak}) and slow walking at ~40% VO_{2peak} for 3 minutes each and ≥4 days/week, during which exercise intensity (energy expenditure per min) was measured with a portable calorimeter equipped with a tri-axial accelerometer and barometer. The purpose of this study is to assess the preventive and therapeutic effects of increased physical fitness by interval walking training on age-associated diseases in middle-aged and older people.

2. Subjects

The subjects were middle-aged and elderly persons, aged 40 to 85 years, participating in a health promotion program for middle-aged and elderly citizens, the "Active Health square by The Matsumoto Physical Training Program for Senior Citizens" project.

3. Criteria for eligibility

Patients who meet all of the following inclusion criteria, but not any exclusion criteria should be regarded as eligible.

3.1. Inclusion criteria

1) Persons, aged 40 to 85 years, participating in a health promotion program for middle-aged and elderly citizens, the "Active Health Square by The Matsumoto Physical Training Program for Senior Citizens" project.
2) Those who can walk independently.

3) Those who have received their home doctors' permission to participate in the study.

4) Those who gave us their informed consents after explained about the details of the study.

3.2. Exclusion criteria

1) Persons who are unable to walk.

2) Those who are judged to be inappropriate for participating the study by the lead principal investigator.

3.3. Subjects in whom informed consent by a legal representative is necessary and its reasons

No corresponding subject.

4. Study methods

4.1. Study design

- Single arm intervention

4.2. Study outline

The protocol of this study is shown below. Physical characteristics, blood pressure, blood lipids and glucose, peak aerobic capacity (VO_{2peak}), thigh muscle strength, CES-D, bone mineral density, and markers for calcium metabolism are measured before and after the IWT regimen.

IWT regimen: Before the start of training, subjects are invited to a community office near their homes and instructed to repeat >5 sets of 3-minute low-intensity walking at ~40% of the pretraining VO_{2peak} for walking followed by 3-minute high intensity at ≥70%VO_{2peak} for ≥4 days/wk. The energy expenditure during daily walking at their favorite time and place is monitored with a triaxial accelerometer (Jukudai Mate; Kissei Comtec, Matsumoto, Japan) on the right or left waist on the midclavicular line. A beeping signal alerts participants when a change of intensity is scheduled.

Every 2 weeks, the participants visit a local office to transfer their walking records from the accelerometer to a central server at the administrative center through the Internet for automatic analysis and reporting. Trainers use these reports to track daily walking intensity and other parameters to instruct participants on how best to achieve the target

levels.

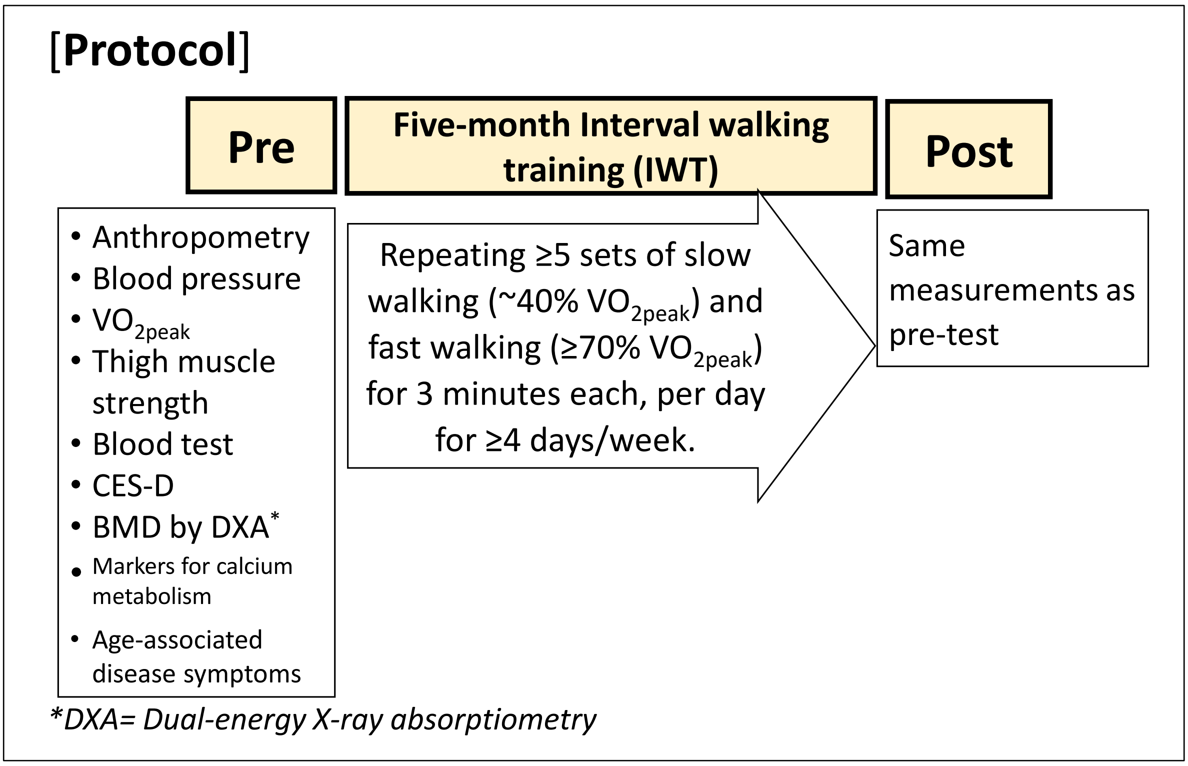

4.4. Regulations for combined drugs (combination therapy)

Subjects under treatment may take drugs during the intervention period. However, drug administration must be recorded.

4.5. Regulations for dose-reduction and discontinuation

Subjects under treatment may take drugs in parallel. However, drug history must be recorded.

4.6. Management of subjects after the completion of this study

After the completion of this study, the study director will provide advice that may be the most adequate, including the outcome obtained in this study, to subjects.

5. Subject assignment

5.1. Subject registration

The study director or coordinators (investigators) should write necessary information in a subject registration sheet, and submit it to a person in charge of registration.

5.2. Assignment methods and assignment-regulating factors

This is not applicable because this study is single-arm intervention.

6. Outcomes

Primary outcomes: Peak aerobic capacity before and after interval walking training

Secondary outcomes: Age-associated disease symptoms; the symptoms of lifestyle-related diseases (hypertension, hyperglycemia, obesity and dyslipidemia), orthopedic diseases (chronic osteoarthritis and osteoporosis), and psychiatric disorders (insomnia, depression, and dementia)

7. Observation/examination items

Measurement schedule

Period / Measurement items	1 week before pre-training assessment	Pre-training assessment	2 weeks after the start of intervention	Every 2 weeks after the start of intervention	5 months after the start of intervention	Post-training assessment
Informed consent	○					
Body Composition Assessment		○				○
Blood pressure		○				○
Physical fitness test		○				○
Blood test		○				○
CES-D		○				○
Bone mineral density		○				○
Markers for calcium metabolism		○				○
Age-associated disease symptoms		○				○
Exercise intensity and volume during IWT			○	○	○	

Transmission of the exercise intensity and volume recorded on a calorimeter			○	○	○	○
Health survey by physicians	△	△	△	△	△	△
Adverse events	△	△	△	△	△	△

○: Only examination is performed, and the results should be reported if necessary.

△: The development of abnormalities or adverse events must be reported.

●: Reporting is necessary.

8. Management of adverse events

8.1. Definition of adverse events

8.1.1. Definition of adverse events

Adverse events were defined as all unfavorable or non-intended disorders or signs (including abnormal laboratory data) in study subjects regardless of the presence or absence of a causal relationship with this study.

8.1.2. Definition of severe adverse events

Severe adverse events (SAEs) refer to adverse events corresponding to one of the following items among adverse events:

1. Fatal adverse events
2. Life-threatening adverse events
3. Adverse events requiring admission for treatment or the prolongation of the admission period
4. Those leading to permanent or marked disorders/dysfunction
5. Those that may induce congenital abnormalities in offspring

8.2. Management of subjects on the appearance of adverse events

The study director or investigators must inform subjects of the appearance of adverse events, and promptly refer them to adequate medical institutions.

8.3. Evaluation of adverse events/reporting

When SAEs occur, the investigators should take necessary measures, such as explanation to subjects, and promptly report them to the study director.

When being informed of the development of an SAE, the study director should promptly prepare "a report on a severe adverse event (1st report)" (Shinshu University School of Medicine clinical research plan Form 4), and report it to the head of the School of Medicine. As a rule, the study director must prepare "a report on a severe adverse event (2nd report)" (Shinshu University School of Medicine clinical research plan Form 5) within 7 days, and report it to the head of the School of Medicine. Furthermore, information on the development of the adverse event should be promptly shared with the investigators involved in this study.

8.4. Expected adverse events

In this study, as an expected adverse event, falling related to high-intensity interval walking may occur although its risk is low.

9. Target number of registered subjects

Target number of registered subjects: 1000

10. Statistical matters

10.1. Reasons for the establishment of the target number of registered subjects

We will verify that the effects of IWT on physical fitness and age-associated diseases. This is an exploratory study, and the data from previous studies are insufficient. The N value to achieve the detection power "significance level on two-sided tests: $\alpha \leq 0.05$, $(1-\beta) \geq 0.8$" is unclear. Therefore, both the α and $(1-\beta)$ values will be described in articles as a reference for additional studies/research.

10.2. Statistical analysis

One-way ANOVA for repeated measures is used to examine any significant changes in the variables after training. To examine any influence of covariant factors on the effects of IWT, we use ANCOVA (analysis for covariance) or multiple regression analysis.

10.3. Analytical items/methods

An outline of statistical analysis is shown below. A p-value of 0.05 is regarded as significant (two-sided test).

10.3.1. Outline of subjects to be analyzed

1) Subjects' background factors and baseline data

Subjects' physical properties and physical fitness before training should be adopted as baseline data.

2) Information on training execution

The number of days when training was performed per week, duration of high-intensity walking per day of training, and duration of slow walking are calculated.

10.3.2. Hypothesis-verifying analysis regarding primary endpoints

After training intervention, increases in physical fitness and improvements of age-associated disease symptoms can be expected.

10.3.3. Analysis regarding accessory endpoints

We expect that after the intervention, increases in physical fitness will be accompanied by improvements in age-associated diseases.

11. Entry/submission of case reports

11.1. Form and deadline for submission

The "case report" form is attached. The deadline for submission is 1 week after the final day of intervention.

11.2. Cautions for filling in case reports

When filling in case reports, please comply with the following matters:

1) Case reports should be written using a black or blue ballpoint pen or pen.
2) In a blank column, a diagonal line should be drawn to distinguish it from an "omission".
3) Before submission, consistency with original materials, such as medical records, must be confirmed.
4) When changing or revising descriptions, the corresponding part should be deleted with

a double line, and correct matters should be written. The investigator responsible for revision must affix a seal or sign the paper with the date.

5) A seal to be used when writing case reports should be unified in each patient.

12. Monitoring

According to the procedure manual for monitoring (including the monitoring plan) prepared by the study director, the monitor should confirm that subjects' human rights, safety, and welfare are protected, that this study is conducted in accordance with the latest protocol, "Ethical Guidelines for Medical and Health Research involving Human Subjects", that the data obtained are accurate and complete, and that they can be verified in the context of relevant records, such as medical records. Actual operations will be conducted by Mayuko Morikawa, Department of Sports Medical Sciences, Shinshu University Graduate School of Medicine.

13. Audit

As this is a single arm exploratory study, an audit will not be performed.

14. Ethical matters

14.1. Regulations to be observed

The persons concerned in this study must observe the "Declaration of Helsinki" by the World Medical Association and "Ethical Guidelines for Medical and Health Research involving Human Subjects".

14.2. Informed consent

The investigators must hand over an explanatory document for obtaining informed consent approved by the Ethics Review Board of our university to subjects, explain the contents using the document and verbally, and obtain written informed consent based on subjects' free will.

When information that may influence subjects' consent is obtained, or when there is a change of the protocol that may influence subjects' consent, the investigators must promptly provide information to subjects, and confirm their will for study participation. In addition, they should revise the explanatory document for obtaining informed consent through approval by the Ethics Review Board of our university, and obtain additional

consent from subjects.

The explanatory document should contain the following contents:

① Study name and permission from the director of a research institute regarding the execution of the corresponding study

② Research institute's name and study director's name (including the names of collaborative research institutes and names of the study directors of these institutes when the study is conducted in collaboration with other research institutes)

③ Purpose and significance of the study

④ Study methods (including the purpose of utilizing samples/information obtained from study subjects) and period

⑤ Reasons for the selection of study subjects

⑥ Subjects' burdens, expected risks/advantages

⑦ Even when subjects agree to study execution or continuation, they can withdraw from the study at any time (when it is difficult to take measures in accordance with the contents of withdrawal from study subjects, the contents and reasons must be explained).

⑧ Even when subjects do not agree to study execution or continuation, or even when they withdraw from the study, there will be no disadvantage for subjects.

⑨ Methods to disclose information on research

⑩ Subjects can obtain or inspect the protocol and materials regarding study methods in a range in which there is no problem for protecting other subjects' personal information and securing the originality of the study based on their requests. Methods to obtain or inspect them.

⑪ Handling of personal information (for anonymization, its methods are included).

⑫ Storage and abolishment of samples/information

⑬ Status of the research-related conflict of interest for research institutes, such as funding sources, and that for investigators, such as personal income

⑭ Management of consultations from the subjects and persons concerned

⑮ Contents of financial burdens or rewards for subjects (if such matters are present)

⑯ Presence or absence and contents of compensations for health damage related to the corresponding study in the case of invasive research

⑰ The samples/information obtained from subjects may be used for future research that is not specified on obtaining informed consent from subjects, or provided to

other research institutes (if there is such a possibility). Contents expected on obtaining informed consent.

⑱ On the assumption that subjects' secrets may be kept, persons engaged in monitoring/audit and review-board members may inspect samples/information regarding the corresponding subjects in a necessary range.

14.3. Protection of personal information

When handling information regarding study execution, a correspondence table should be prepared by attaching a number that is not related to the subject's personal information, and anonymization should be conducted so that subjects' secrets may be protected. The correspondence table must be strictly managed by a personal information administrator, and should not be provided to external organizations. When publishing the study results, attention must be paid so that information that allows the identification of subjects may not be included. Furthermore, the subject information obtained in the study should not be used for purposes other than the study purpose.

14.4 Storage of samples/information

Information (materials) regarding this study (documents on research and study data) must be stored in a lockable storage apparatus in the Shinshu University Graduate School of Medicine for 10 years after outcome presentation, such as article publication, under the direction of a person responsible for management (study director). After the completion of the storage period, anonymized paper data should be discarded using a shredder. Electronic data should be completely deleted.

15. Recording matters regarding the provision of samples/information

As there is no transfer of samples/information, records will not be prepared.

16. Change of the study protocol

If the protocol of this study or explanatory document for obtaining informed consent is changed or revised, this must be initially approved by the Ethics Review Board of our university.

17. Research expenses

17.1. Research funds and conflict of interest

This study will be conducted with the grant from the Ministry of Health, Labor and Welfare of Japan. Furthermore, investigators participating in this study must report necessary matters to the industry-academia collaboration conflict of interest management committee of Shinshu University according to the “Shinshu University School of Medicine procedures for ethical review application” so that the contents may be inspected and approved.

17.2. Subjects’ cost allocation or rewards

This study will be supported by the grant above and participants will not pay any costs for this study.

17.3. Management of health damage and compensations

If health damage occurs in subjects during this study, investigators must provide adequate treatment. Furthermore, compensations for health damage should be paid according to the “Ethical Guidelines for Medical and Health Research involving Human Subjects”. There may be no fatal, severe adverse events; therefore, there will be no problem if insurance is not established. Thus, in this study, no monetary compensations for subjects’ health damage are prepared. This will be approved by the Ethics Review Board of our university, and sufficiently explained to subjects. We will ask them to participate in this study based on their understanding and consent.

18. Study period and the completion/early discontinuation of this study

18.1. Study period

Subject registration period: Date of approval–September 30, 2009

Study period: Date of approval–March 31, 2010

18.2. Completion of this study

When measurement is completed, this study will be completed. The study director will promptly submit a report on study completion to the head of the School of Medicine.

18.3. Early discontinuation of this study

The investigators should examine whether study execution/continuation is possible when the following matters are met.

1) When it is considered extremely difficult to reach a target number of patients due to difficulties in subject incorporation
2) When the study purpose is achieved before a target number of patients or intended period is reached
3) When there may be problems regarding safety based on safety information such as reports on severe adverse events
4) When adequate study continuation is considered to be difficult due to deviations from the ethical guidelines/study protocol or contract violations
5) When it is considered difficult to accept a direction for a change of the protocol from the Ethics Review Board of our university

The study director should discontinue this study if discontinuation is recommended or directed by the Ethics Review Board of our university. If study discontinuation is determined, the study director must promptly report this and its reasons in writing to the head of the School of Medicine.

19. Storage of medical instruments, usage, and storage period

Personal management will be thoroughly conducted by attaching a number so that there may be no misuse, reforming, or misunderstanding of a portable calorie meter to be delivered to each subject.

20. Storage of records

The study director must store important documents regarding study execution (a copy of the application form, notifications from the hospital director, copies of various applications/reports, informed consent, and other documents or records necessary to assure the data reliability) for 10 years after outcome presentation, such as article publication. Subsequently, these documents should be discarded, paying attention to personal information.

21. Attribution of the study outcome

The outcome of this study belongs to Shinshu University. The study director will publish

the outcome of this study through presentations at relevant society meetings.

22. Study execution system

This study will be conducted by the following system:

[Study director]

Professor, Hiroshi Nose, Shinshu University Graduate School of Medicine (Study arrangement)

[Investigators]

Mayuko Morikawa, Department of Sports Medical Sciences, Shinshu University Graduate School of medicine (Study execution)

Shizue Masuki, Department of Sports Medical Sciences, Shinshu University Graduate School of Medicine (Study execution)

[Personal information administrator]

Shunichi Furuhata, Jukunen Taiikudaigaku Research Center (JTRC), Health promotion coordinator

[Data administrator]

Professor, Hiroshi Nose, Shinshu University Graduate School of Medicine

[Person responsible for registration]

Professor, Keiichi Higuchi, Shinshu University Graduate School of Medicine

[Person in charge of monitoring]

Mayuko Morikawa, Department of Sports Medical Sciences, Shinshu University Graduate School of medicine

[Contact information] Inquiries regarding the study contents

Study bureau: Professor Hiroshi Nose

Department of Sports Medical Sciences, Shinshu University Graduate School of Medicine

3-1-1, Asahi, Matsumoto City 390-8621, Japan

TEL: +81+263-37-2682, FAX: +81-263-34-6721

23. References

1. Blair SN, Kohl HW, 3rd, Paffenbarger RS, Jr., Clark DG, Cooper KH, and Gibbons LW. Physical fitness and all-cause mortality. A prospective study of healthy men and women. *JAMA* 262: 2395-2401, 1989.

2. Manson JE, Greenland P, LaCroix AZ, Stefanick ML, Mouton CP, Oberman A, Perri MG, Sheps DS, Pettinger MB, and Siscovick DS. Walking compared with vigorous exercise for the prevention of cardiovascular events in women. *N Engl J Med* 347: 716-725, 2002.

3. King AC, Haskell WL, Young DR, Oka RK, and Stefanick ML. Long-term effects of varying intensities and formats of physical activity on participation rates, fitness, and lipoproteins in men and women aged 50 to 65 years. *Circulation* 91: 2596-2604, 1995.
