# Supplementary material for: Increased response of postmenopausal bone to interval walking training depends on baseline bone mineral density
Source: PLoS One. 2024 Sep 5;19(9):e0309936. doi: 10.1371/journal.pone.0309936 (PMC11376574; doi:10.1371/journal.pone.0309936)
Supplement: S2 Protocol — (PDF) [file pone.0309936.s003.pdf]

（信州大医臨床研究計画様式）

信州大学医学部臨床研究計画書

提出日	平成 17 年 6 月 5 日
提出番号＊	
諾否決定日＊	
諾否＊	
承認番号＊	

＊申請者においては記入しないこと

以下、項目番号を変更しないこと

1. 計画名		松本市熟年体育大学・いきいき健康ひろば事業参加者を対象とした運動処方効果の検証
2. 分類		■① 単独グループによる研究<br>□② 多施設共同研究の主任研究者<br>□③ 多施設共同研究への参加［他施設の倫理委員会での承認証明書を添付のこと］<br>□④ その他（
3. 参照すべき倫理指針		■①「臨床研究に関する倫理指針」<br>□②「疫学研究に関する倫理指針」<br>□③ なし（理由 ）
4. 研究機関名・研究実施場所		信州大学大学院医学研究科・スポーツ医科学分野
5. 研究責任者氏名（所属・職名）		能勢 博（信州大学大学院医学研究科・教授）
6. 研究者等氏名（所属・職名）		森川真悠子（信州大学大学院医学研究科・大学院生）
7. 共同臨床研究機関名（役割）		なし
8. 被験者	(1) 被験者の選定方針及び予想される被験者数	松本市熟年体育大学いきいき健康ひろば事業参加者中高年男女合わせて 1,000 人
	(2) 被験者に死者、未成年者、または判断能力の不十分な成人は含まれるか	なし
9. 研究の意義・目的<br>＊別紙添付の場合も本欄に概要を記入すること		超高齢社会において、中高年者の健康増進のため、インターバル速歩の体力向上と生活習慣病・骨粗鬆症をはじめとする加齢性疾患の予防効果を検証し、新しい運動処方の指針を開発する。
10. 研究方法<br>＊別紙添付の場合も本欄に概要を記入すること		松本市「熟年体育大学」事業に参加する自立歩行可能な年齢 40-85 歳の男女を対象に、インターバル速歩、携帯型カロリー計（熟大メイト）、インターネット端末からなる遠隔型個別運動処方システムを用いて数カ月の運動指導介入を行い、介入中の運動の強度と量、介入前後で体力、血液成分、骨密度などの測定を行う。インターバル速歩とは、個人の最高酸素摂取量の４０％の強度のゆっくり歩きと 70%以上の早歩きを３分間ずつ交互に繰り返す歩行方法である。
11. 研究期間		2005 年承認日から 2010 年 3 月 31 日（最大５年）
12. 当該臨床研究に参加することにより期待される利益及び起こりうる危険並びに必然的に伴う不快な状態<br>＊別紙添付の場合も本欄に概要を記入すること		利益：体力向上、生活習慣病を含む加齢性疾患症状の改善効果<br>研究に伴う危険または不快な状態：ウォーキング中の転倒事故など

13. 当該臨床研究後の対応<br>＊別紙添付の場合も本欄に概要を記入すること	提供された情報は、研究終了後に破棄する。ただし、新たな研究に際して被験者より得た情報を使用して解析する場合、本人から同意を得てこれを行う。この場合も同様に被験者個人の情報は次項に従い保護される。
14. 当該臨床研究に係る個人情報保護の方法（被験者を特定できる場合の取り扱いを含む）<br>＊別紙添付の場合も本欄に概要を記入すること	「資料の保管」資料の収集、管理などの扱いにあたって個人情報の漏洩、盗難」紛失等が起きないように、資料の匿名化、不必要資料の破棄、資料管理責任の徹底を図る。<br>「資料の公表」本人に通知する場合を除き、資料収集に関して、目的、責任者を定め、開示等に関する事項については公表する。<br>「開示・訂正請求」資料の個人情報に関し、本人からの「開示」の求めがあった場合には、それを拒否する合理的理由がない限り、申し出た本人に対してのみ開示する。また、資料の個人情報の「訂正」が本人から求められた場合も、これに従う。<br>「研究結果の発表」研究結果を学会や学術誌で外部に公表する場合には、被験者の匿名化を行い、データの連結解析を不可能にする。
15. インフォームド・コンセントのための手続き	■① 被験者からインフォームド・コンセントを得て研究を行う<br>[→16 に必ず記載のこと]<br>□② 代諾者等からインフォームド・コンセントを得て研究を行う<br>[→18 に必ず記載のこと]<br>□③ インフォームド・コンセントを得ないで研究を行う<br>□④ その他（　　　　　　　　　　　　）
16. 代諾社からインフォームド・コンセントを得て研究を行う場合<br>＊別紙添付の場合も本欄に概要を記入すること	[当該臨床研究の重要性]<br><br>[被験者が研究を実施するにあたり必要不可欠な場合]<br><br>[代諾者等の選定方針]
17. インフォームド・コンセントを得るための説明文書記載事項<br>＊別紙添付の場合も本欄に概要を記入すること	■①当該臨床研究への参加は任意であること<br>■②当該臨床研究への参加に同意しないことをもって不利益な対応を受けないこと<br>■③被験者または代諾者等は、自らが与えたインフォームド・コンセントについて、いつでも不利益を受けることなく撤回することができる。<br>■④被験者として選定された理由<br>■⑤当該臨床研究の意義、目的、方法及び期間<br>■⑥研究者等の氏名及び職名<br>■⑦予測される当該臨床研究の結果、当該臨床研究に参加することにより期待される利益及び起こりうる危険並びに必然的に伴う不快な状態、当該臨床研究終了後の対応<br>■⑧被験者及び代諾者の希望により、他の被験者の個人情報保護や該当臨床研究の独創性の確保に支障のない範囲内で、当該臨床研究計画及び当該臨床研究方法についての資料を入手又は閲覧することができること<br>■⑨個人情報の取扱い、提供先の機関名、提供先における利用目的が妥当であること等について倫理審査委員会で審査した上で、当該臨床研究の結果を他の機関へ提供する可能性があること<br>■⑩当該臨床研究の成果により特許権等が生み出される可能性があること及び特許権等が生み出された場合の帰属先<br>■⑪被験者を特定できないようにした上で、当該臨床研究の成果が公表される可能性があること<br>■⑫当該臨床研究に係る資金源、起こりうる利害の衝突及び研究者等の関連組織との関わり[1]<br>■⑬当該臨床研究に伴う補償[2]の有無（当該臨床研究に伴う補償があるばあいにあっては、当該補償の内容を含む。）

		■⑭問い合わせ、苦情糖の窓口の連絡等に関する情報<br>□⑮被験者からインフォームド・コンセントを受けることが困難な場合は、当該臨床研究の重要性及び被験者の当該臨床研究への参加が当該臨床研究を実施するに当たり必要不可欠な理由<br>□⑳その他（　　　　　　　　　　　　　　　　）
18. インフォームド・コンセントを得ないで研究を行う場合	(1)研究対象	□①個人を特定できない人由来の材料及びデータのみを用いる<br>□②個人を特定できる人由来の材料及びデータを用いる[→(2)へ]
	(2)(1)=②の場合、使用する材料及びデータの種類	□①研究開始前に人体から採取された材料（試料）を用いる[→18(5)②の措置を講じること]<br>□②人体から採取された材料（試料）を用いず、既存のデータのみを用いる[→(5)①の措置を講ずること]<br>□③人体から採取された材料（試料）を用いず、新たに採集したデータを用いる[→18(5)②の措置を講ずること]<br>□④その他(　　　　　　　　　　　　　　　　　　)
	(3)研究の性質	①当該研究が被験者に対する最小限の危険を超える危険を含むか<br>□含む<br>□含まない（その理由:　　　　　　　　　　）<br>②インフォームド・コンセントを免除することが被験者の不利益となるか<br>□不利益となる<br>□不利益とならない<br>③インフォームド・コンセントを免除しなければ、実際上、当該研究を実施できず、または当該研究の価値を著しく損ねるか<br>□実施できる/研究の価値が損なわれない<br>□実施できない/研究の価値が損なわれる(その理由：　　　　)<br>④当該研究が社旗的に重要性の高いものであると認められるか<br>□認められる（その理由：　　　　　　　　　　　　）<br>□認められない
	(4)インフォームドコンセントに代わる措置の有無	□　①あり[→(5) へ]<br>□　②なし
	(5)(4)=①の場合、インフォームド・コンセントに代わる措置の内容	□①被験者が含まれる集団に対し、資料の収集・利用の内容を、その方法も含めて広報する<br>□②研究の実施についての情報を公開し、研究対象者となるものが研究対象者となることを拒否できるようにする<br>□③できるだけ早い時期に、被験者に事後的説明（集団に対するものも可）を与える<br>□④長期間にわたって継続的に資料が収集又は利用される場合には、社会に、その実情を、資料の収集又は利用の方法も含めて広報し、社会へ周知される努力を払う<br>□⑤その他（具体的に　　　　　　　　　　　）
19. 当該臨床研究に係る資金源		■①文部科学省等の公的研究費<br>□②その他（具体的に：　　　　　　　　　　）
20. 起こりうる利害の衝突		□①起こりうる（情報を開示する）<br>■②起こりえない
21. 当該研究に伴う補償		■①あり（　　　　　　　　　　　　　　　　）<br>□②なし（　　　　　　　　　　　　　　　　）
22. 確認事項		■①研究責任者に、臨床研究を適正に実行するための必要な専門的知識及び臨床経験が十分あること<br>■②健康に影響を与えるような行為を伴う人を対象とする臨床研究（いわゆる介入研究）の場合には、臨床経験が十分にある医師による適切な助言を得ていること[医師名：信州大学大学院医学研究科教授　能勢　博]

	■③研究者等は、臨床研究を実施するに当たっては、一般的に受け入れられた科学的原則に従い、科学的文献その他科学に関連する情報源及び十分な実験に基づいていること．<br>□④研究者等は、環境に影響を及ぼすおそれのある臨床研究を実施する場合又は臨床研究の実施に当たり動物を使用する場合には、十分な配慮をしていること．<br>■⑤臨床研究機関の長が重篤な有害事象その他の臨床研究の適正性及び信頼性を確保するための調査を行う場合には研究責任者は、これに必要な情報を報告すること．<br>□⑥研究責任者は、他の臨床研究機関と共同で臨床研究を実施する場合には、当該他の臨床研究機関の研究責任者に対し、臨床研究に起因する重篤な有害事象を報告すること．<br>■⑦研究責任者は、臨床研究により期待される利益よりも起こりうる危険が高いと判断される場合又は臨床研究により十分な成果が得られた場合には、当該臨床研究を中止し、又は終了すること．<br>■⑧研究責任者は、臨床研究を終了するまでの間、臨床研究に関する国内外における学会発表、論文発表等の情報（以下「発表情報等」という．）について把握しておくとともに、把握した当該発表情報等について、臨床研究機関の長に対し、報告すること．<br>□⑨研究責任者は、他の臨床研究機関と共同で臨床研究を実施する場合には、当該他の臨床研究機関の研究責任者に対し、把握した発表情報等について報告すること．<br>■⑩研究責任者は、臨床研究を中止し、又は終了した場合には、その旨を臨床研究機関の長へ報告すること．<br>■⑪研究責任者は、臨床研究を実施するに当たり、被験者の個人情報の保護のために必要な措置を講じていること．<br>■⑫研究者等は、臨床研究の結果を公表する場合には、被験者を特定できないように行うこと．<br>■⑬研究責任者は、臨床研究終了後においても、被験者が当該臨床研究の結果により得られた最善の予防、診断及び治療を受けることができるよう努めること．<br>□⑭その他
22. その他記載すべき事項	

1「利害の衝突」とは，研究者等が研究の実施や報告の際に，金銭的な利益やそれ以外の個人的な利益のためにその専門的な判断を曲げてしまう（もしくは曲げたと判断される）ような状況や，当該研究の実施によって本務である教育・研究・診療等に支障をきたすような状況を示す．（前者を狭義の「利益相反」，後者を「責務相反」という．）

この狭義の「利益相反」は，金銭的な利害の衝突とそれ以外の利害の衝突に分類できる．

金銭的な利害の衝突とは、研究者等が資金提供や研究依頼のあった者・団体（政府，財団，企業等）から臨床研究に係る資金源の他に機器や消耗品等の提供を受けること，実施料を受け取ること，その株式を所有（未公開株やストックオプションを含む）すること，特許権を共有・譲渡されること，講演料や著述料の支払いを受けていること等である．

それ以外の利害の衝突とは，研究者等が資金提供や研究依頼のあった者・団体との間に顧問等の非常勤を含む雇用関係があることや，親族や師弟関係等の個人的関係があることなど，研究者等の関連組織との関わりについての問題などが考えられる．（臨床研究に関する倫理指針Q& Aより）

上記の様な利害の衝突の中でも，明示的に確認することができる①資金源等の金銭上の利害の衝突②研究者等の関連組織との関わりについては少なくとも記載するべきである．

2「補償」とは、臨床研究の実施により被験者が当該臨床研究で不利益になる有害事象が生じた場合に、研究者側に過失がなくとも健康被害に係る医療費その他の費用を研究者側が支払うものを指す。（臨床研究に関する倫理指針Q& Aより）
