# Supplementary material for: Increased response of postmenopausal bone to interval walking training depends on baseline bone mineral density
Source: PLoS One. 2024 Sep 5;19(9):e0309936. doi: 10.1371/journal.pone.0309936 (PMC11376574; doi:10.1371/journal.pone.0309936)
Supplement: S1 File — (DOCX) [file pone.0309936.s004.docx]

**Increased response of postmenopausal bone to interval walking training depends on baseline bone mineral density**

Rizka Nugraheni Martyanti, Mayuko Morikawa, Masaaki Hanaoka, Satoshi Tanaka, Yukio Nakamura, Hiroshi Nose, Shizue Masuki

**Supplementary Methods and Results**

**Methods**

**Analyses and statistics**

**Confirmation of independent factors influencing baseline BMDs**

There were several cross-sectional studies showing that some items of physical fitness and health status influence BMDs [1-6]. In the present study, to confirm the factors independently associated with baseline BMD, we performed multiple regression analyses for each bone site. For the first step of the analyses, we entered into the stepwise model the variables considered for independent determinants, which were the baseline physical characteristics, LSD score, physical activity (**Table 1**). Additionally, we entered alcohol intake, smoking, and health status (**Table 2**) as dichotomous variables (applicable=1, not applicable=0) for each subject. As a result, candidate determinants identified by the analyses were body mass index (BMI), isometric knee extension force (*F*_EXT_), and physical activity for baseline BMDs for all bone sites, and age for baseline BMD for FN and TH. Then, as the final step of the analyses, we entered these variables into the forced entry model (**Supplementary** **Table S3**).

**Results**

**Confirmation of independent factors influencing baseline BMDs**

**Supplementary** **Table S3** shows the results of the multiple regression analysis for baseline BMD for each bone site. We found that BMI and physical activity were significant independent determinants of baseline BMDs for all bone sites (all, P<0.04). In addition, age and *F*_EXT_ were significant independent determinants of baseline BMDs for FN and TH (all, P<0.01).

**References**

1. Whalen RT, Carter DR, Steele CR. Influence of physical activity on the regulation of bone density. J Biomech. (1988) 21:825-37. doi: 10.1016/0021-9290(88)90015-2.
2. Madansingh SI, Ngufor CG, Fortune E. Quality over quantity: skeletal loading intensity plays a key role in understanding the relationship between physical activity and bone density in postmenopausal women. Menopause. (2020) 27:444-449. doi: 10.1097/GME.0000000000001486.
3. LeBoff MS, Greenspan SL, Insogna KL, Lewiecki EM, Saag KG, Singer AJ, et al. The clinician's guide to prevention and treatment of osteoporosis. Osteoporos Int. (2022) 33:2049-2102. doi: 10.1007/s00198-021-05900-y.
4. Masugata H, Senda S, Inukai M, Murao K, Hosomi N, Iwado Y, et al. Association between bone mineral density and arterial stiffness in hypertensive patients. Tohoku J Exp Med. (2011) 223:85-90. doi: 10.1620/tjem.223.85.
5. Sugimoto T, Sato M, Dehle FC, Brnabic AJ, Weston A, Burge R. Lifestyle-related metabolic disorders, osteoporosis, and fracture risk in Asia: a systematic review. Value Health Reg Issues. (2016) 9:49-56. doi: 10.1016/j.vhri.2015.09.005.
6. Saddik H, Pinti A, Antoun A, Al Rassy N, El Hage Z, Berro AJ, et al. Limb muscular strength and bone mineral density in elderly subjects with low skeletal muscle mass index. J Clin Densitom. (2021) 24:538-547. doi: 10.1016/j.jocd.2021.03.011.
