# Supplementary material for: Increased response of postmenopausal bone to interval walking training depends on baseline bone mineral density
Source: PLoS One. 2024 Sep 5;19(9):e0309936. doi: 10.1371/journal.pone.0309936 (PMC11376574; doi:10.1371/journal.pone.0309936)
Supplement: S1 Table — (DOCX) [file pone.0309936.s005.docx]

| **Supplementary Table S1. Changes in physical fitness and LSD score** **from baseline after the intervention for identifying independent factors to increase BMDs by IWT** | | | | | |
| --- | --- | --- | --- | --- | --- |
| Variable |  | LS | | FN | |
|  | All  (n=234) | Lower  (n=64) | Higher  (n=170) | Lower  (n=80) | Higher  (n=154) |
| ΔBody mass, kg | 0.3±0.1 | 0.5±0.1 | 0.3±0.1 | 0.4±0.1 | 0.3±0.1 |
| ΔVO_2peak_, L/min | -0.01±0.01 | -0.01±0.02 | -0.01±0.01 | -0.01±0.02 | -0.01±0.01 |
| ΔHR_peak_, beats/min | 1.4±1.2 | 0.3±2.5 | 1.8±1.3 | -1.0±2.3 | 2.6±1.3 |
| Δ*F*_EXT_, Nm | 3.5±1.0 | 2.9±1.7 | 3.8±1.3 | 3.4±1.6 | 3.6±1.4 |
| Δ*F*_FLX_, Nm | -0.5±0.7 | -0.6±1.5 | -0.5±0.8 | -1.4±1.3 | -0.1±0.9 |
| ΔLSD score | 0.13±0.05 | 0.17±0.10 | 0.11±0.06 | 0.14±0.09 | 0.12±0.06 |
| Values are mean ± SE. LSD score, lifestyle-related disease score; BMD, bone mineral density; IWT, interval walking training; LS, lumbar spine; FN, femoral neck; Lower, subjects with lower baseline BMD; Higher, subjects with higher baseline BMD; ∆, the change after the exercise intervention; VO_2peak_, peak aerobic capacity for walking; HR_peak_, peak heart rate; *F*_EXT_, isometric knee extension force; *F*_FLX_, isometric knee flexion force. | | | | | |
