# Supplementary material for: Increased response of postmenopausal bone to interval walking training depends on baseline bone mineral density
Source: PLoS One. 2024 Sep 5;19(9):e0309936. doi: 10.1371/journal.pone.0309936 (PMC11376574; doi:10.1371/journal.pone.0309936)
Supplement: S2 Table — (DOCX) [file pone.0309936.s006.docx]

**Supplementary Table S2. Markers for calcium metabolism before and after the intervention for examining any influence on the differences in ΔBMDs between the lower and higher BMD groups**

| Variable | All (n=65) | LS | | FN | |
| --- | --- | --- | --- | --- | --- |
|  |  | Lower (n=11) | Higher (n=54) | Lower (n=24) | Higher (n=41) |
| High-sensitivity PTH, pg/ml |  |  |  |  |  |
| Pre | 361.7±11.1 | 389.1±22.5 | 356.1±12.5 | 375.0±20.5 | 353.9±12.9 |
| Post | 348.9±12.6 | 400.9±34.1 | 338.3±13.1 | 375.0±22.1 | 333.7±14.8 |
| ∆ | -12.8±6.8 | 11.8±24.1 | -17.8±6.5 | 0.0±11.3 | -20.2±8.4 |
| Calcitonin, pg/ml |  |  |  |  |  |
| Pre | 25.2±1.7 | 29.3±7.1 | 24.4±1.5 | 26.4±3.8 | 24.5±1.6 |
| Post | 25.3±1.4 | 27.5±5.5 | 24.9±1.3 | 25.9±3.1 | 25.0±1.3 |
| ∆ | 0.1±0.8 | -1.7±2.4 | 0.4±0.9 | -0.5±1.5 | 0.4±1.0 |
| 25(OH)D, ng/ml |  |  |  |  |  |
| Pre | 20.6±0.4 | 19.3±0.9* | 20.8±0.4 | 19.3±0.6* | 21.3±0.4 |
| Post | 20.8±0.4 | 19.4±0.9* | 21.1±0.4 | 19.7±0.7* | 21.5±0.5 |
| ∆ | 0.2±0.4 | 0.1±1.2 | 0.3±0.4 | 0.4±0.7 | 0.1±0.5 |
| Values are mean ± SE. BMD, bone mineral density; LS, lumbar spine; FN, femoral neck; Lower, subjects with lower baseline BMD; Higher, subjects with higher baseline BMD; PTH, parathyroid hormone; 25(OH)D, 25-hydroxyvitamin D; ∆, the change after the exercise intervention. *Significant differences from the high group, P<0.05. | | | | | |
