# Supplementary material for: Increased response of postmenopausal bone to interval walking training depends on baseline bone mineral density
Source: PLoS One. 2024 Sep 5;19(9):e0309936. doi: 10.1371/journal.pone.0309936 (PMC11376574; doi:10.1371/journal.pone.0309936)
Supplement: S3 Table — (DOCX) [file pone.0309936.s007.docx]

| **Supplementary Table S3. The results of multiple regression analysis for confirming independent factors influencing the baseline BMDs before the intervention** | | | | | | | | | | | |
| --- | --- | --- | --- | --- | --- | --- | --- | --- | --- | --- | --- |
| Variable | Baseline LS | | |  | Baseline FN | | |  | Baseline TH | | |
|  | β (95% CI) | Std β | *P* value |  | β (95% CI) | Std β | *P* value |  | β (95% CI) | Std β | *P* value |
| Age, yr | -29 x 10^-4^ (-70 x 10^-4^ to 10 x 10^-4^) | -0.10 | 0.14 |  | -61 x 10^-4^ (-87 x 10^-4^ to -35 x 10^-4^) | **-0.28** | **<0.001** |  | -57 x 10^-4^ (-84 x 10^-4^ to -30 x 10^-4^) | **-0.25** | **<0.001** |
| BMI, kg/m² | 0.012 (0.0064 to 0.018) | **0.25** | **<0.001** |  | 0.010 (0.0059 to 0.014) | **0.28** | **<0.001** |  | 0.014 (0.010 to 0.018) | **0.38** | **<0.001** |
| *F*_EXT_, Nm | 6.9 x 10^-4^ (-0.54 x 10^-4^ to 14 x 10^-4^) | 0.12 | 0.069 |  | 6.8 x 10^-4^ (1.8 x 10^-4^ to 12 x 10^-4^) | **0.17** | **0.007** |  | 6.7 x 10^-4^ (1.6 x 10^-4^ to 12 x 10^-4^) | **0.16** | **0.009** |
| Physical activity | 0.035 (0.0022 to 0.068) | **0.13** | **0.036** |  | 0.027 (0.0048 to 0.048) | **0.14** | **0.017** |  | 0.037 (0.014 to 0.059) | **0.18** | **0.001** |
| BMD, bone mineral density; LS, lumbar spine; FN, femoral neck; TH, total hip; BMI, body mass index; *F*_EXT_, isometric knee extension force; β, unstandardized coefficient; Std β, standardized coefficient. Values in boldface indicate significant determinants. The analysis was performed on all subjects (n=234). | | | | | | | | | | | |
